# The EGF Domains of MUC4 Oncomucin Mediate HER2 Binding Affinity and Promote Pancreatic Cancer Cell Tumorigenesis

**DOI:** 10.3390/cancers13225746

**Published:** 2021-11-16

**Authors:** Nicolas Stoup, Maxime Liberelle, Céline Schulz, Sumeyye Cavdarli, Romain Vasseur, Romain Magnez, Fatima Lahdaoui, Nicolas Skrypek, Fabien Peretti, Frédéric Frénois, Xavier Thuru, Patricia Melnyk, Nicolas Renault, Nicolas Jonckheere, Nicolas Lebègue, Isabelle Van Seuningen

**Affiliations:** 1 Univ. Lille, CNRS, Inserm, CHU Lille, UMR9020-U1277—CANTHER—Cancer Heterogeneity Plasticity and Resistance to Therapies, F-59000 Lille, France; nicolas.stoup@inserm.fr (N.S.); celine.schulz@univ-lille.fr (C.S.); sumeyye.cavdarli@univ-lille.fr (S.C.); romain.vasseur87@gmail.com (R.V.); romain.magnez@inserm.fr (R.M.); fatima.lahdaoui@univ-lille.fr (F.L.); nicolas.skrypek@gmail.com (N.S.); peretti.fabi@gmail.com (F.P.); frederic.frenois@chru-lille.fr (F.F.); xavier.thuru@inserm.fr (X.T.); nicolas.jonckheere@inserm.fr (N.J.); 2 Univ. Lille, Inserm, CHU Lille, U1172—LilNCog—Lille Neurosciences & Cognition, F-59000 Lille, France; maxime.liberelle@univ-lille.fr (M.L.); patricia.melnyk@univ-lille.fr (P.M.); 3 Univ. Lille, Inserm, CHU Lille, U1286—INFINITE—Institute for Translational Research in Inflammation, F-59000 Lille, France; nicolas.renault@univ-lille.fr

**Keywords:** HER2, MUC4, protein–protein interaction, pancreatic cancer, EGF domain

## Abstract

**Simple Summary:**

A feature of pancreatic cancer (PC) is the frequent overexpression of tyrosine kinase membrane receptor HER2 along with its membrane partner the MUC4 oncomucin in the early stages of the pancreatic carcinogenesis. However, therapeutic approaches targeting HER2 in PC are not efficient. MUC4 could indeed represent an alternative therapeutic strategy to target HER2 signaling pathway, but this approach needs to characterize MUC4/HER2 interaction at the molecular level. In this study, we successfully showed the impact of the EGF domains of MUC4 on HER2 binding affinity and demonstrated their “growth factor-like” biological activities in PC cells. Moreover, homology models of the MUC4_EGF_/HER2 complexes allowed identification of binding hotspots mediating binding affinity with HER2 and PC cell proliferation. These results allow a better understanding of the mechanisms involved in the MUC4/HER2 complex formation and may lead to the design of potential MUC4/HER2 inhibitors.

**Abstract:**

The HER2 receptor and its MUC4 mucin partner form an oncogenic complex via an extracellular region of MUC4 encompassing three EGF domains that promotes tumor progression of pancreatic cancer (PC) cells. However, the molecular mechanism of interaction remains poorly understood. Herein, we decipher at the molecular level the role and impact of the MUC4_EGF_ domains in the mediation of the binding affinities with HER2 and the PC cell tumorigenicity. We used an integrative approach combining in vitro bioinformatic, biophysical, biochemical, and biological approaches, as well as an in vivo study on a xenograft model of PC. In this study, we specified the binding mode of MUC4_EGF_ domains with HER2 and demonstrate their “growth factor-like” biological activities in PC cells leading to stimulation of several signaling proteins (mTOR pathway, Akt, and β-catenin) contributing to PC progression. Molecular dynamics simulations of the MUC4_EGF_/HER2 complexes led to 3D homology models and identification of binding hotspots mediating binding affinity with HER2 and PC cell proliferation. These results will pave the way to the design of potential MUC4/HER2 inhibitors targeting the EGF domains of MUC4. This strategy will represent a new efficient alternative to treat cancers associated with MUC4/HER2 overexpression and HER2-targeted therapy failure as a new adapted treatment to patients.

## 1. Introduction

**Figure 1:**
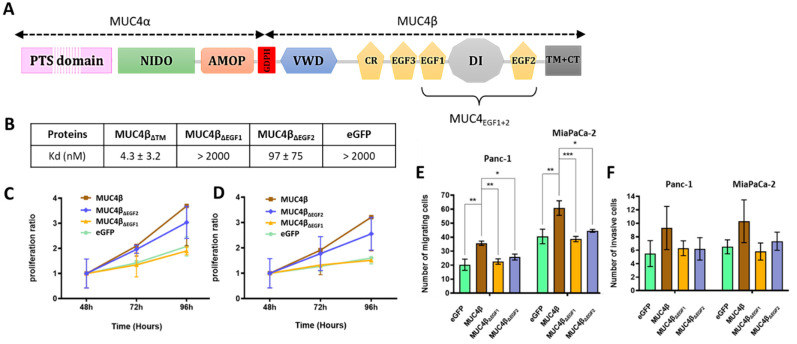
Impact of deletion of MUC4_EGF1_ and MUC4_EGF2_ domains on binding affinities with HER2 and on proliferation and migration properties of human PC cells. (**A**) Schematic representation of the membrane-bound mucin MUC4 featuring both subunits MUC4α and MUC4β. MUC4α is rich in proline/serine/threonine residues (PST domain), is strongly *O*-glycosylated, and contains the AMOP and the NIDO domains. The subunits are post-transcriptionally cleaved by an auto-cleavable GDPH sequence and noncovalently associated. MUC4β is formed by a von Willebrand factor-D (VWD), an uncharacterized strongly *N*-glycosylated part, a cysteine-rich domain (CR), two EGF-like domains (EGF3 and EGF1), an intermediate uncharacterized domain (DI), another EGF-like domain (EGF2), and a transmembrane helix (TM) with a short cytoplasmic tail (CT). (**B**) Measurement of binding affinities between HER2 and eGFP-MUC4β_ΔTM_ containing the EGF domains (MUC4β_ΔTM_) and the deleted forms for each EGF domain: eGFP-MUC4β_ΔEGF1_ (MUC4_ΔEGF1_), eGFP-MUC4β_ΔEGF2_ (MUC4_ΔEGF2_), or eGFP alone (mock, negative control), using MST. Measurement of Panc-1 (**C**) and MiaPaCa-2 (**D**) cell proliferation after transfection with eGFP-MUC4β containing the EGF domains and the deleted forms for each domain: MUC4β_ΔEGF1_, MUC4β_ΔEGF2_, or eGFP alone (negative control). Proliferation ratio over eGFP was calculated for each construct. Measurement of cell migration with Boyden chamber (control insert) (**E**) and cell invasion with Boyden chamber (Matrigel^®^ insert) (**F**) in Panc-1 and MiaPaCa-2 cells after transfection with eGFP-MUC4β, the deleted forms MUC4β_ΔEGF1_ and MUC4β_ΔEGF2_, or eGFP alone (negative control). Statistically significant differences between eGFP-MUC4β and the deleted forms of MUC4 or eGFP alone are indicated; * *p* < 0.05, ** *p* < 0.01, and *** *p* < 0.001.

The MUC4 membrane-bound mucin is one of the largest multimodular glycoproteins at the cell surface characterized by two noncovalent subunits. MUC4α is the highly glycosylated mucin-type extracellular subunit, while MUC4β is the transmembrane (TM) subunit that contains several functional domains, including three epidermal growth factor (EGF)-like domains followed by a short cytoplasmic tail (Figure 1A). MUC4 is overexpressed in many epithelial cancers and extensively regarded as a pro-tumorigenic protein as it is not expressed in healthy pancreas, while is neoexpressed as early as the pancreatic preneoplastic stage [1,2,3,4,5]. MUC4 expression in cancer is also associated with poor prognosis of the tumor and poorer overall survival of patients, which makes it a potent prognostic biomarker [6]. The HER2 receptor belongs to the epidermal growth factor receptor (EGFR/ErbB) family of receptor tyrosine kinases and represents one of the main oncogenes involved with aggressiveness and poor prognosis of cancers [7]. Although no soluble ligand has been identified for HER2, TM mucins were shown to interact with HER2 leading to cell proliferation, migration, invasion, and resistance to apoptosis [8]. Among the TM mucins/HER complexes, the interaction of MUC4 with HER2 has drawn a lot attention since they are both overexpressed at the PC cell surface, forming an oncogenic complex participating in cancer cell tumorigenic properties and in activation of different oncogenic signaling pathways leading to tumor progression [1,3,9,10]. HER2 targeting in cancers is currently following two approaches: (i) targeted therapies using monoclonal antibodies preventing HER2 interaction, or (ii) tyrosine kinase inhibitors blocking phosphorylation of HER2, thereby inhibiting its downstream signaling activity [11,12]. These approaches, even though they show patient response at the beginning of the treatment, rapidly develop resistance, which inevitably leads to disease progression and death [13,14]. Moreover, in some cancers, they remain inefficient [15]. Targeting the MUC4β part of the TM mucin via protein–protein interaction (PPI) inhibitors has, thus, become an alternative route to target MUC4/HER2 overexpressing cancers [16], as well as to rescue the targeting of HER2 positive cancers for which direct HER2 targeting has failed [17] or triggered strong secondary effects [18]. A big challenge in this approach will be to overcome the fact that MUC4 is heavily glycosylated and creates a steric hindrance that has already been shown to hamper access of therapeutic antibodies targeting HER2 [19,20,21,22]. In the past few years, we started to decipher the molecular mechanism of interaction between MUC4 and HER2 and revealed the druggability of the PPI interface as a promising therapeutic target [8,23,24]. In addition to showing that endogenous MUC4 directly interacts with HER2 in PC cell lines (Supplementary Materials Figure S1A,B), we quantified the binding affinity between MUC4β and HER2 using microscale thermophoresis and showed that the interaction is mediated by the three EGF domains [25]. The present study involves the structure–function relationship between MUC4_EGF_ domains and HER2 at the molecular level and their biological activity on human pancreatic cancer cells, confirming the potency for developing small inhibitory molecules targeting MUC4_EGF_ domains with therapeutic value.

## 2. Material and Methods

### 2.1. Cell Lines and Cell Culture

MiaPaCa-2, Panc-1, and Panc-89 human pancreatic and 647-V human bladder cancer cells were purchased from American Type Culture Collection (ATCC, Manassas, VA, USA) and were grown as previously described [26]. MUC4-KD and mock cell lines were generated as in [27]. Total cell extracts and protein measurement were carried out as previously described [10].

### 2.2. Generation of GST-EGF Fusion Proteins

The wild-type EGF sequences were amplified by PCR from the pCDNA3(+) vector containing MUC4β sequence and subsequently cloned into the pGEX-4-T1 vector (GE Healthcare Life Sciences, Buc, France) as previously described [10] to generate GST-EGF1, GST-EGF2, and GST-EGF1+2 constructs. Site-directed mutagenesis was realized using the Quick Change II site-directed mutagenesis kit according to the manufacturer’s instructions (Agilent Technologies, Santa Clara, CA, USA). Each mutated aa was mutated to an Ala residue. Primer sequences are shown in Table S1. After sequencing, constructs were transfected in *E. coli* strain B834(DE3)pLysS (Novagen, Merck Millipore, Darmstadt, Germany), and the GST fusion proteins were produced by induction with 1 mM isopropyl-β-d-thiogalactopyranoside (Ambion™, Invitrogen, Thermo Fisher Scientific, Waltham, MA, USA) at 15 °C overnight. Bacteria were centrifuged, and the pellet was lysed by sonication at 4 °C using Bioruptor^®^ Plus (Diagenode, SA, Seraing, Belgium) in lysis buffer (1× phosphate-buffered saline pH 7.4 (1× PBS)) containing EDTA-free protease inhibitor cocktail (Roche, Basel, Switzerland) and 1.2 µg/mL of deoxyribonuclease I (Euromedex, Souffelweyersheim, France). Centrifuged supernatant was then incubated with equilibrated Glutathione Sepharose 4B resin (GE healthcare Life Sciences, Buc, France) in 1× PBS for 3 h at 4 °C, and GST-fusion proteins were eluted with 50 mM Tris-HCl, pH 8.0 buffer containing 20 mM of reduced glutathione (Sigma-Aldrich, Merck Millipore, Darmstadt, Germany). An aliquot of the purified proteins was separated by SDS-PAGE on a NuPAGE™ 4–12% acrylamide Bis/Tris gel (Novex, Thermo Fisher Scientific, Waltham, MA, USA) and stained with R250 Coomassie^®^ blue (Serva, Heidelberg, Germany). The fusion proteins to be used in functional assays were then dialyzed at 4 °C in 1× PBS buffer using a centrifugal filter unit with a 10 kDa cutoff (Amicon^®^ Ultra 0.5 mL, Merck Millipore, Darmstadt, Germany). Protein concentration was determined at 280 nm using NanoDrop^®^ 1000 spectrophotometer (Thermo Scientific, Waltham, MA, USA).

### 2.3. Generation and Activity of eGFP-MUC4β, eGFP-MUC4β_ΔTM_, and eGFP-MUC4_ΔEGFs_ Fusion Proteins

To generate soluble eGFP-MUC4 fusion proteins, the MUC4 sequence from Uniprot (accession number #Q99102) was used. In order to obtain a monomeric eGFP-MUC4β fusion protein (from #I1447-#P2169, optimized for CHO-K1 expression), only the β subunit was kept, except for the four aa involved in the cleavage (GDPH) (ProteoGenix SAS, Schiltigheim, France). The corresponding DNA sequence was cloned into the peGFP-C1 vector using the appropriate restriction sites, with kanamycin and neomycin resistance genes. The constructs were synthesized by e-Zyvec company (Lille, France). Two series of eGFP mutants were produced. Soluble mutants (Supplementary Figure S2A) were devoid of the TM (transmembrane) and CT (cytoplasmic tail) domains of MUC4, and included purification tags, eGFP and cleavable linker: eGFP-MUC4β_ΔTM_ (lacks TM, CT): #I1447-#K2122; eGFP-MUC4β_ΔEGF1_ (lacks TM, CT and EGF1): #I1447-Δ(#F1872-#F1920)-#K2122; eGFP-MUC4β_ΔEGF2_ (lacks TM, CT, and EGF2): #I1447-#C2077. Membrane-bound mutants (Supplementary Figure S2B) included the β-subunit of MUC4 (#I1447-#P2169) with TM and CT domains and the native signal peptide of MUC4 (#M1-#G28): full (MUC4β) or were devoid of either EGF1 (ΔEGF1) or EGF2 (ΔEGF2) domain. These mutants also bear purification tags and eGFP. Expression, purification of constructs, and transfection followed by cell lysate preparation were performed as described before [25,28]. Titration was carried out by fluorescence using the NanoTemper Monolith NT.115 instrument [25]. For in vitro studies, Panc-1 and MiaPaCa-2 cells were grown to 65–70% confluency in DMEM (Gibco, Thermo Fisher Scientific) for 72 h and then 24 h in Opti-MEM (Gibco, Thermo Fisher Scientific) prior to transfection. Cells were then transfected with 3 µg of eGFP-MUC4 constructs (e-Zyvec, Lille, France) using the Lipofectamine 3000 transfection kit according to the manufacturer’s protocol (Thermo Fisher Scientific). Cell proliferation and migration assays were performed at least in triplicate as described thereafter.

### 2.4. GST Pull-Down Assay

The GST pull-down assay was performed as previously described [10] with some modifications: Pellets from 50 mL of bacterial culture were sonicated at 4 °C using Bioruptor^®^ Plus (Diagenode) in lysis buffer (1× PBS, 1 mM EDTA, 0.1% Triton X-100 (*v*/*v*), 1 mg/mL lysozyme) containing EDTA-free protease inhibitor cocktail (Roche). GST-pull down assays were performed at least in triplicate.

### 2.5. Immunoprecipitation of HER2

HER2 immunoprecipitation (IP) was performed as previously described [10]. IP proteins were then separated and immunostained as described thereafter.

### 2.6. SDS-PAGE and Western-Blotting

Protein samples from total cell extracts (20 µg), GST pull-down, and immunoprecipitation assays were mixed with 4× SDS loading buffer and boiled at 100 °C for 5 min before analysis on a NuPAGE™ 3–8% acrylamide Tris-acetate gel (Novex, Thermo Fisher Scientific). Western blotting was carried out on a PVDF membrane (0.45 µm Immobilon^®^-P, Merck Millipore, Darmstadt, Germany). Antibodies used for interaction and cell signaling pathway studies are listed in Table S2. Membranes were then incubated with peroxidase-conjugated secondary antibodies (Pierce), and the signal was visualized using the West Pico chemiluminescent substrate (Thermo Fisher Scientific). Chemiluminescence was detected using Image Quant LAS 4000 apparatus (GE Healthcare Life Sciences). Intensity of bands was quantified and integrated using Image Quant TL 8.1 software (GE Healthcare Life Sciences). Electrophoresis, transfer, and Western blotting conditions for MUC4 expression were as described in [29]. All original uncropped western blot can be found in Figure S13.

### 2.7. PLA Assay

In situ proximity ligand assays in MUC4-KD and mock cells were performed using Duolink In Situ Red Starter Kit (Sigma Aldrich, Merck Millipore, Darmstadt, Germany) following the manufacturer’s protocol as described before [10].

### 2.8. MUC4_EGFs_/HER2 Interaction Studies Using Microscale Thermophoresis

MST experiments were conducted with the Monolith NT.115 instrument using the optimized method previously described [25]. Briefly, experiments were conducted either by following the native fluorescence of eGFP-fusion proteins for lysate titration against HER2 or by tagging HER2 for titration of GST-fusion proteins. The following proteins were used: recombinant HER2-Fc (R&D Systems, Inc., Minneapolis, MN, USA), HER2-Fc-like domain-containing protein PD1-Fc (R&D Systems, Inc., Minneapolis, MN, USA), eGFP-MUC4β (Supplementary Materials Figure S2A,B), and nonrelevant eGFP-PD1 fusion protein as a negative control. All assays were performed in triplicate with two distinct cell lysates (*n* = 6).

### 2.9. Cell Proliferation Assay

Cells were seeded at 1.5 × 10^5^ cells per well in six-well plates into a medium containing 2% (*v*/*v*) FCS and treated or not with MUC4_EGF_ recombinant proteins as indicated. Cells were counted at 24, 48, 72, and 96 h using a Malassez counting chamber using Trypan Blue exclusion dye (Invitrogen, Thermo Fisher Scientific, Waltham, MA, USA). Treatments were repeated every day. Assays were performed at least in triplicate.

### 2.10. Cell Migration and Invasion Assays

MiaPaCa-2 and Panc1 cells were serum-starved 24 h before experimentation in cell culture medium containing 2% FCS (*v*/*v*). Then, 1 × 10^5^ cells were seeded for 24 h in the upper chamber of a 24-well Boyden chamber without (migration) or with Matrigel^®^ (Corning^®^ 354262, Glendale, AZ, USA) (invasion), whereas the lower chamber was filled with medium containing 2% (*v*/*v*) FCS with or without MUC4_EGF_ recombinant proteins. Membranes between the two chambers were then recovered and stained with mounting medium with DAPI (Vectashield, Vectorlabs, Burlingame, CA, USA). Staining was visualized with a Zeiss LSM 710 confocal microscope (Zeiss, Jena, Germany), images were captured and analyzed with the Zeiss Efficient Navigation software (Zeiss, Jena, Germany). Assays were performed at least in triplicate.

### 2.11. Computational Methods: Homology Modeling of Human MUC4_EGF1_/HER2 and MUC4_EGF2_/HER2C Complexes

The human sequences of HER2, MUC4_EGF1_, MUC4_EGF2_ (P04626, Q99102 Uniprot IDs), hEGF, and EGFR (extracted from the 1IVO PDB structural template) were aligned using ClustalΩ [30]. Although MUC4_EGF1_ and MUC4_EGF2_ domains correspond to regions 1875–1914 and 2078–2117, respectively, in the full annotated sequence of human MUC4 (Q99102 Uniprot reference), their C-terminal region was extended with eight aa to fit with the whole-paired sequence of the hEGF structural template. This initial alignment was manually refined using Chimera [31] to adjust some of the gaps in the loop regions and align all conserved cysteine residues (Supplementary Materials Figure S4A). Using this alignment, MUC4_EGF1_/HER2 and MUC4_EGF2_/HER2 complex structures were modeled using the Modeller program [32] with the crystal structure of hEGF-ErbB1 (IVO PDB entry) as a template. Residues missing in the template were refined using the loop optimization method in Modeller, and disulfide bridges were added between 42 paired cysteine residues of HER2 on one hand and between six paired cysteine residues of EGF1 or EGF2 on the other hand (Supplementary Materials Figure S4B). All models were subjected to 300 iterations of variable target function method optimization and thorough molecular dynamics (MD) and simulated annealing optimization, and they were scored using the discrete optimized protein energy potential. The five best-scoring models were inspected visually and mapped to a Ramachandran diagram; the most suitable model of each complex was selected in terms of low score and structure of the loops (Supplementary Materials Figure S5A).

### 2.12. Molecular Dynamics (MD) Simulations

After two successive steps of steepest descent and conjugate gradient energy minimization, both systems were equilibrated by MD simulations restraining α-carbons at the desired temperature of 300 K during 100 ps in a NVT (constant N particles, volume, and temperature ensemble) with a velocity rescaling thermostat at the desired pressure of 1 bar during 300 ps in a NPT (constant N particles, pressure, and temperature) ensemble with an isotropic Parinello–Rahman coupling. The full energy relaxation of both complex models was achieved by triplicate of 100 ns unrestrained MD simulations in an NPT ensemble with water molecules as the explicit solvent and using the CHARMM forcefield [33] implemented in GROMACS 5.1.2 software [34]. MD simulations were performed using a time step of 2 fs while constraining all bonds between hydrogen and heavy atoms by the P-LINCS algorithm [35].

### 2.13. Proteome Array Studies

Panc-1 and MiaPaCa-2 cells were grown to 80% confluency and then serum-starved for 24 h in culture medium containing 2% FCS (*v*/*v*). Cells were then treated for 5 min with the GST-EGF domains or GST alone at 1 µg/mL, with hEGF at 25 ng/mL, or untreated. Proteome arrays were performed according to the manufacturer’s instructions with 300 µg of total protein extract from each condition using the human Phospho Kinase array or XL ONCO array kits (Proteome Profiler™, R&D system Inc., Minneapolis, MN, USA). Chemiluminescence was detected using Image Quant LAS 4000 (GE Healthcare Life Sciences). Signal visualization and quantification were carried out as above. Assays were performed in duplicate with two dots per protein (*n* = 4).

### 2.14. Subcutaneous Xenografts in Scid Mice

MiaPaCa-2 or Panc-1 cells (2 × 10^6^ cells in 100 µL) were subcutaneously injected with 100 µL of Matrigel^®^ (Corning^®^ 354262) into flanks of male Severe Combined Immunodeficient (SCID) mice (six per group) aged 6 weeks (CB17, Charles Rivers, Ecully, France). Tumor size evaluation and follow-up was performed as before [36]. Injections (100 µL) were performed twice a week with 50 µg of GST-MUC4_EGF_ domains (2 mg/kg) or 25 ng of hEGF (50 µg/kg), as a positive control, per mouse. Moreover, 1× PBS and GST alone (50 µg, 2 mg/kg) were also injected as negative controls. High amounts of GST-MUC4_EGF1_ and GST-MUC4_EGF2_ recombinant proteins required for in vivo experiments were produced and purified by Proteogenix SAS (Schiltigheim, France). All procedures were in accordance with the guideline of animal care committee (protocol #00423.02, Comité Ethique Expérimentation Animale Nord Pas-de-Calais, CEEA75).

### 2.15. Statistics

Statistical analyses were performed with GraphPad Prism4 software (GraphPad Software Inc., La Jolla, CA, USA). Data are presented as the mean ± SEM. Differences in the mean of samples were analyzed by one-way ANOVA with selected comparisons using Tukey’s test or by two-way ANOVA with selected comparisons using Bonferroni post hoc test, and the differences were considered significant at *p* < 0.05 *, *p* < 0.01 **, or *p* < 0.001 ***.

## 3. Results

### 3.1. MUC4_EGF1_ and MUC4_EGF2_ Drive the Binding Affinity with HER2 and Mediate Both Cell Proliferation and Migration of Human Pancreatic Cancer Cells

In our recent work [8], we described that the minimal sequence of MUC4 to interact with HER2 encompasses the region from EGF1 to EGF2 domains. We also showed that these EGF domains display distinct behavior as HER2 ligands instead of being just part of one bigger domain. To go further in the characterization, we then generated eGFP-MUC4β fusion proteins and their mutants in which a single EGF domain was deleted at a time (Supplementary Materials Figure S2A,B). Structure–function studies were performed to identify which of the EGF domains was essential for driving binding affinity with HER2 and for mediating proliferation and migration of cancer cells. We analyzed MUC4/HER2 interactions using microscale thermophoresis (MST) as previously described [25]. In the MST studies, each construct was deleted from the TM part of MUC4β in order to produce soluble proteins (Supplementary Materials Figure S2A). The protein lacking a TM domain (MUC4β_ΔTM_) displayed a Kd value of 4.3 ± 3.2 nM. The effects of the deletions on the binding affinities with HER2 clearly showed the central role of EGF1 since the protein lacking EGF1 (MUC4β_ΔEGF1_) led to a total loss of affinity (Kd > 2000 nM), similar to the mock negative control (Kd > 2000 nM) (Figure 1B and Supplementary Materials Figure S3A). Deletion of EGF2 (MUC4β_ΔEGF2_) only led to a decrease in the binding affinity with a Kd value of 97 nM. When using the MUC4β protein and the MUC4β_∆EGF1/∆EGF2_ mutants addressed to the membrane (Supplementary Materials Figure S2B) in MUC4 non-expressing Panc1 and MiaPaCa-2 PC cells, the data clearly indicated that removal of EGF1 abrogated all proliferative activity of MUC4β with complete decrease in activation and return to baseline (eGFP), whereas removal of EGF2 had a partial effect (Figure 1C,D). The effects on cell migration (Figure 1E) and invasion (Figure 1F) were similar for both mutants, with an almost complete decrease in activation and return to baseline (equivalent to eGFP) when EGF domains were absent. These results support our initial hypothesis describing the interaction between MUC4 and HER2 as modular and involving both EGF1 and EGF2 domains. MUC4_EGF1_ appears central for both binding affinity with HER2 and activation of the oncogenic signaling pathways leading to proliferation, while MUC4_EGF2_ displays partial effects. This suggests that both domains could be carrying distinct activities or at least be part of a multi-epitope binding site. Thus, we next focused on MUC4_EGF1_ and MUC4_EGF2_ domains and explored their molecular characteristics.

### 3.2. MUC4_EGFs_ Domains Physically Interact with HER2

**Figure 2:**
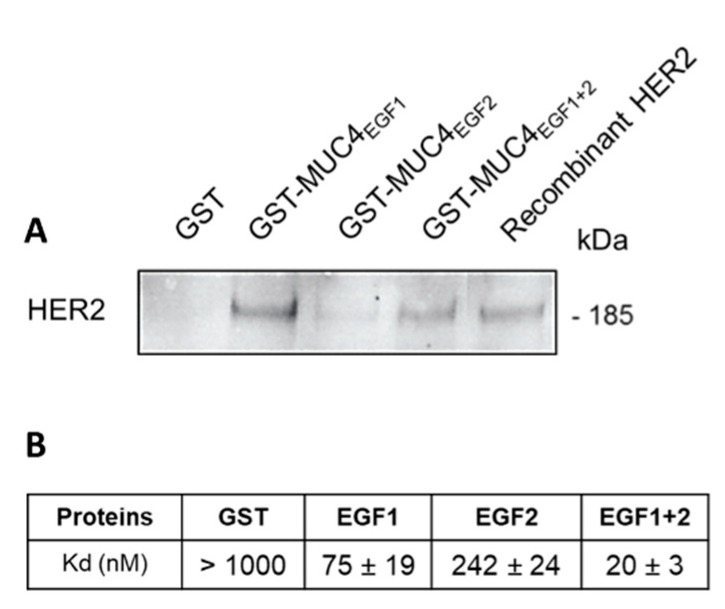
MUC4_EGFs_ domains physically interact with HER2 in vitro. (**A**) Immunoblot with anti-HER2 antibody of GST pull-down assays of recombinant HER2 with GST alone, GST-MUC4_EGF1_, GST-MUC4_EGF2_, and GST-MUC4_EGF1+2_. Recombinant HER2 alone was used as control. (**B**) Measurement of binding affinities between fluorescent tagged HER2 and GST-MUC4_EGF1_ (EGF1), GST-MUC4_EGF2_ (EGF2), GST-MUC4_EGF1+2_ (EGF1+2), or GST alone (negative control), using MST.

In order to show that MUC4_EGFs_ domains are involved in direct interaction with HER2, we produced MUC4_EGF1_, MUC4_EGF2_, and MUC4_EGF1+2_ domains as GST fusion proteins, as previously described [10]. We first showed by GST-pull down that MUC4_EGF1+2_ and MUC4_EGF1_ domains were able to directly interact with HER2 (Figure 2A). MUC4_EGF2_ showed a weaker interaction. Binding affinity measurements using MST also supported the central role of MUC4_EGF1_ with a Kd value of 75 ± 19 nM, while MUC4_EGF2_ displayed a Kd of 242 ± 24 nM. The MUC4_EGF1+2_ combination module resulted in a better affinity (Kd = 20 ± 3 nM), close to the Kd obtained with the full MUC4β subunit (Figure 2B and Supplementary Materials Figure S3B). This result confirms that the MUC4_EGF1+2_ region resumes most of the binding potency of MUC4β for HER2.

### 3.3. Molecular Dynamics (MD) Simulations of MUC4_EGF1_/HER2 and MUC4_EGF2_/HER2 Complex Models Led to Virtual Binding Hotspot Identification

**Figure 3:**
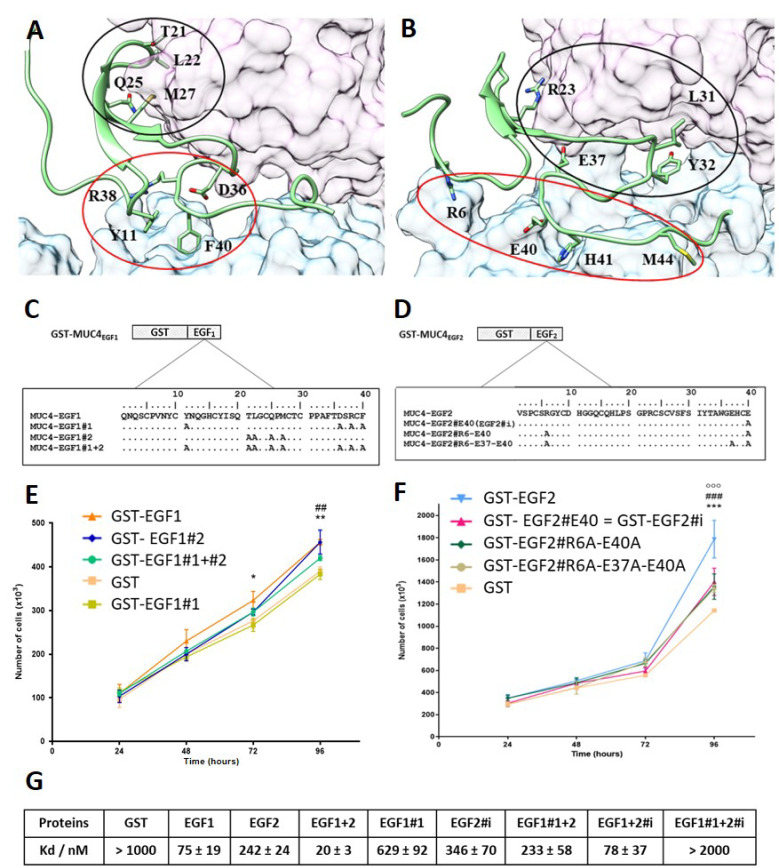
MD simulations of MUC4_EGF1_/HER2 and MUC4_EGF2_/HER2 complex models, hotspot identification, and effects of mutations. Intermolecular interactions within (**A**) MUC4_EGF1_/HER2 and (**B**) MUC4_EGF2_/HER2 complexes after MD simulations. The molecular surface maps of domains I and III of HER2 are shown in pink and cyan, respectively. MUC4_EGF_ domains appear in green, and cluster 1 and cluster 2 are surrounded in red and black, respectively. The illustration was made using UCSF Chimera v1.10.2. The aa sequences of wt and mutated GST-MUC4_EGF1_ (**C**) and GST-MUC4_EGF2_ (**D**) domains. The aa residues identified by MD as key binding hotspots were mutated into an alanine residue as indicated. EGF1#1: mutation of Y11, D36, R38, and F40; EGF1#2: mutation of T21, L22, Q25, and M27; EGF1#1 + #2: mutation of Y11, T21, L22, Q25, M27, D36, R38, and F40; EGF2#R6-E37-E40: mutation of R6, E37, and E40; EGF2#R6-E40: mutation of R6 and E40; EGF2#E40 (EGF2#i): mutation of E40. (**E**) Measurement of Panc1 cell proliferation after cell treatment with wild-type GST-EGF1, GST-EGF1#1, GST-EGF1#2, and GST-EGF1#1 + #2 at 1 µg/mL. * Statistically significant difference between GST-EGF1 and GST-EGF1#1; ^#^ statistically significant difference between GST-EGF1#2 and GST-EGF1#1. (**F**) Measurement of Panc-1 cell proliferation after cell treatment with wild-type GST-EGF2, GST-EGF2#R6A-E37A-E40A, GST-EGF2#R6A-E40A, and GST-EGF2#E40A (EGF2#i) at 1 µg/mL. Statistically significant differences between GST-EGF2 and GST-EGF2#i are indicated; * *p* < 0.05, **^, ##^ *p* < 0.01, and ***^, ###, ooo^ *p* < 0.005. (**G**) Measurement of binding affinities between fluorescent tagged HER2 and GST-MUC4_EGF1_ (EGF1), GST-MUC4_EGF2_ (EGF2), GST-MUC4_EGF1+2_ (EGF1+2), GST-MUC4_EGF1#1_ (EGF1#1), GST-MUC4_EGF2#i_ (EGF2#i), GST-MUC4_EGF1#1+2_ (EGF1#1+2), GST-MUC4_EGF1+2#i_ (EGF1+2#i), GST-MUC4_EGF1#1+2#i_ (EGF1#1+2#i), or GST alone (negative control), using MST.

*Drosophila* EGFR (dEGFR) and HER2 are described to adopt a similar autoinhibited conformation different from those found in other mammalian ErbB family members [37]. However, previous studies showed that growth factor ligands break autoinhibitory interactions between domains I and III of dEGFR forming a complex structurally homologous to hEGF/hEGFR’s [38,39,40]. We, therefore, suggested that the complex formation between MUC4_EGFs_ domains and HER2 may be similar to that of hEGF/EGFR complex. In the lack of an experimentally established MUC4_EGFs_/HER2 crystal structure, 3D homology modelling of both MUC4_EGF1_/HER2 and MUC4_EGF2_/HER2 complexes benefited from the high sequence homology (59%) with the crystallized hEGF/EGFR complex [40]. This strong similarity comes mainly from the homology between EGFR and HER2 sequences (63% and 44% for homology and identity sequence rates, respectively) whereas conservation across MUC4_EGF1_, MUC4_EGF2_, and hEGF sequences is around 40% homology and 25% identity (Supplementary Materials Figure S4C). Nevertheless, conservation of the three disulfide bridges within each MUC4_EGF_ domain is in favor of their 3D folding (Supplementary Materials Figure S5B), and their low sequence homology is in favor of high specificity for each ErbB family of receptor [8,23]. MD simulations were then undertaken to build 3D MUC4_EGF1_/HER2 and MUC4_EGF2_/HER2 models (Supplementary Materials Figure S5A). The most frequent intermolecular interactions (salt bridges, hydrophobic or aromatic contacts, and polar hydrogen bonds) at the interface of the complexes were monitored and considered as the virtual binding hotspots (Supplementary Materials Figure S6A,B) [41,42]. In each final 3D interface model (Figure 3A,B), domains I, II, and III of the HER2 receptor were arranged in a C shape, and MUC4_EGF_ domains were accommodated between domains I and III in a similar way as hEGF binding to EGFR.

Two clusters of binding residues, designated as cluster 1 (red circle) and cluster 2 (black circle), which interacted with extracellular domains III and I of HER2, respectively, were identified for each modeled complex and revealed a major specificity upon each protein–protein interface. For the MUC4_EGF1_ interface, cluster 1 is essentially constituted by ionic and aromatics contacts (Y11, D36, R38, and F40), stronger than the H-bond and hydrophobic contacts of cluster 2 (T21, L22, Q25, and M27) (Supplementary Materials Figure S6C, left panel). For the MUC4_EGF2_ interface, both clusters display the same types of interactions: ionic (R6, R23, E37, R38, and E40), aromatic (Y32 and H41), and hydrophobic contacts (I31 and M44) (Supplementary Materials Figure S6C, right panel).

### 3.4. Cluster Mutation Decreases In Vitro Proliferation of Human Pancreatic Cancer Cells and Binding Affinity of the Mutated MUC4_EGF_ Domains

To experimentally validate the binding hotspots, we generated mutants in which each aa identified as participating in the interaction was mutated to an Ala residue (Figure 3C,D) [43]. Proliferation studies in Panc-1 human PC cells showed that mutation of cluster 1 of MUC4_EGF1_ (GST-EGF1#1) domain induced a statistically significant decrease in proliferation at 72 h that persisted at 96 h. Mutation of cluster 2 (GST-EGF1#2) was not as potent despite a decrease in proliferation as well (Figure 3E). Therefore, in the remainder of the manuscript, only the EGF1#1 quadruple mutant of cluster 1 was used. For the MUC4_EGF2_ domain, since some of the aa of EGF1#1 concentrating the maximum affinity for HER2 were of ionic nature, we focused the mutations only on aa involved in salt bridges (R6, E37, and E40). Proliferation studies clearly showed that the R6A/E37A/E40A triple mutant (GST-EGF2#R6A-E37A-E40A) induced a statistically significant decrease in proliferation at 96 h. Interestingly, the change of the triple mutant to the E37A/E40A (GST-EGF2#E37A-E40A) double mutant and E40 (GST-EGF2#i, i for ionic interaction) single mutant showed a similar decrease in proliferation (Figure 3F). This indicates that the E40A mutation is sufficient. Thus, in the remainder of the work, we used the EGF2#i mutant. MST analysis also supported the central role of the hotspots in the binding affinity measurements between GST-MUC4_EGF_ mutants and HER2 (Figure 3G and Supplementary Materials Figure S7). Mutation of cluster1 on MUC4_EGF1_ (EGF1#1) clearly decreased its binding ability (Kd = 629 ± 92 nM) and resulted in a loss of affinity in the EGF1#1+2 module (Kd = 233 ± 58 nM). This mutant displayed almost the same affinity as the single MUC4_EGF2_ domain (Kd = 242 ± 24 nM). Mutation of ionic cluster of MUC4_EGF2_ (EGF2#i) resulted in a slight loss of affinity for the single MUC4_EGF2_ domain (Kd = 346 ± 70 nM) and raised the affinity of the EGF1+2#i module to a Kd value of 78 ± 37 nM, close to that of the single MUC4_EGF1_ domain (Kd = 75 ± 19 nM). The double mutant (EGF1#1+2#i), as expected, did not show any binding affinity. These results validate cluster 1 of MUC4_EGF1_ and ionic cluster of MUC4_EGF2_ as crucial binding hotspots in mediating binding affinity of MUC4 to HER2 and activation of proliferation.

### 3.5. MUC4_EGF_ Domains and Their Mutants Affect In Vitro Proliferation, Migration, and Invasion Properties of Human Pancreatic Cancer Cells

**Figure 4:**
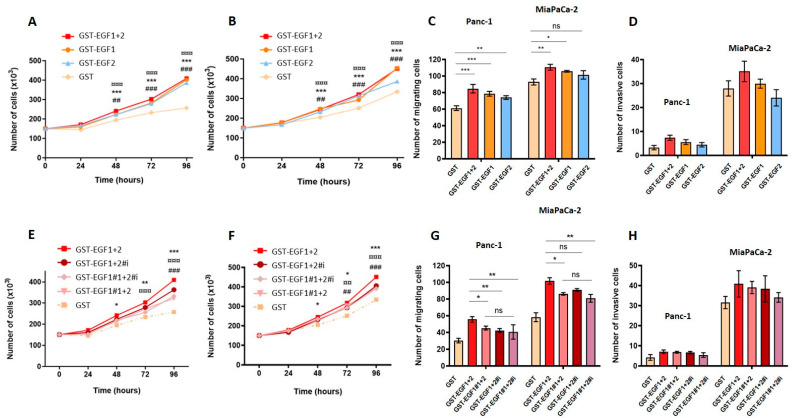
Effects of MUC4_EGF1_ and MUC4_EGF2_ domains on in vitro proliferation, migration, and invasion of human pancreatic cancer cells. Measurement of cell proliferation in Panc-1 (**A**) and MiaPaCa-2 (**B**) cells after treatment with GST-MUC4_EGF1_, GST-MUC4_EGF2_, GST-MUC4_EGF1+2_, or GST alone (negative control) at 1 µg/mL. (**A**,**B**): * Statistically significant difference between GST-MUC4_EGF1_ and GST; ^#^ statistically significant difference between GST-MUC4_EGF2_ and GST; ^¤^ statistically significant difference between GST-MUC4_EGF1+2_ and GST. Measurement of cell migration with Boyden chamber (control insert) (**C**) and cell invasion with Boyden chamber (Matrigel^®^ insert, Corning, Glendale, AZ, USA) (**D**) in Panc-1 and MiaPaCa-2 cells after 24 h of treatment with GST-MUC4_EGF1_, GST-MUC4_EGF2_, GST-MUC4_EGF1+2_, or GST alone at 1 µg/mL. (**C**,**D**): Statistically significant differences between GST-MUC4_EGF1_, GST-MUC4_EGF2_, or GST-MUC4_EGF1+2_ and GST are indicated; * *p* < 0.05, **^, ##^ *p* < 0.01, and ***^, ###, ¤¤¤^ *p* < 0.001. Measurement of cell proliferation in Panc-1 (**E**) and MiaPaCa-2 (**F**) cells after treatment with GST-MUC4_EGF1+2_, the mutated forms GST-MUC4_EGF1#1+2_, GST-MUC4_EGF1+2#i_, and GST-MUC4_EGF1#1+2#i_, or GST alone at 1 µg/mL. (**E**,**F**): Statistically significant differences between GST-MUC4_EGF1+2#1_ and GST-MUC4_EGF1+2_ (*), between GST-MUC4_EGF1+2#i_ and GST-MUC4_EGF1+2_ (^#^), and between GST-MUC4_EGF1#1+2#i_ and GST-MUC4_EGF1+2_ (^¤^) are indicated. Measurement of cell migration with Boyden chamber (control insert) (**G**) and cell invasion with Boyden chamber (Matrigel^®^ insert) (**H**) in Panc-1 and MiaPaCa-2 cells after 24 h of treatment with GST-MUC4_EGF1+2_, GST-MUC4_EGF1#1+2_, GST-MUC4_EGF1+2#i_, GST-MUC4_EGF1#1+2#i_, or GST alone at 1 µg/mL. (**G**,**H**): Statistically significant differences between the mutated forms GST-MUC4_EGF1#1+2_, GST-MUC4_EGF1+2#i_, GST-MUC4_EGF1#1+2#i_ and the wild type GST-MUC4_EGF1+2_ are indicated; * *p* < 0.05, **^, ##, ¤¤^ *p* < 0.01, and ***^, ###, ¤¤¤^ *p* < 0.001. ns: non significant.

We then undertook to determine whether the modulation of the binding potency of MUC4_EGF_ domains would result in a modulation of their “growth factor-like” biological activities. MUC4 non-expressing Panc-1 and MiaPaCa-2 cells were treated with either wild-type domains (Figure 4A,D) or mutated forms (Figure 4E,H) and monitored for the cell proliferation, migration, and invasion. The results clearly show that MUC4_EGF1_, MUC4_EGF2_, and MUC4_EGF1+2_ significantly activate both cell proliferation (Figure 4A,B), with an activation similar to hEGF (Supplementary Materials Figure S8), and cell migration (Figure 4C). Activation of cell invasion was more moderate and did not reach significance (Figure 4D). Mutations in cluster 1, ionic cluster, or both clusters of MUC4_EGF1+2_ significantly reduced proliferation (Figure 4E,F) and migration (Figure 4G) of both cell lines and had no significant effect on cell invasion (Figure 4H). While MUC4_EGF1_ as a single domain appears to carry more biological activity than MUC4_EGF2_, mutation of either cluster 1 or the ionic cluster on the MUC4_EGF1+2_ module provided the same impact on cell proliferation and migration. These results suggest that both single domains could effectively act as alternative ligands of the ErbB family of receptor, with MUC4_EGF1_ more active than MUC4_EGF2_. However, in the MUC4_EGF1+2_ module, both domains seemed to produce a concerted and synergistic effect, with MUC4_EGF1_ more involved in the binding affinity and MUC4_EGF2_ in the oncogenic signaling pathways.

### 3.6. MUC4_EGF_ Domains Enhance Pancreatic Tumor Growth In Vivo

**Figure 5:**
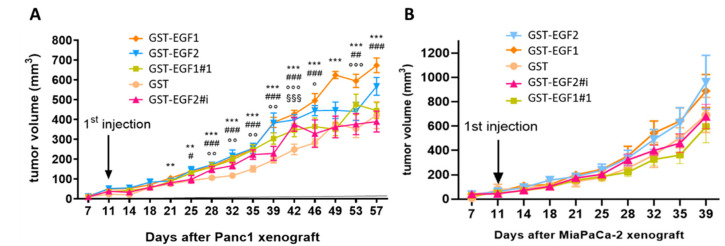
Impact of wild-type or mutated forms of MUC4_EGF1_ and MUC4_EGF2_ domains on human pancreatic tumor growth in vivo. Panc-1 (**A**) or MiaPaCa-2 (**B**) cells were injected with Matrigel^®^ subcutaneously into flank of male Severe Combined Immunodeficient (SCID) mice (*n* = 6) aged 6 weeks (CB17, Charles Rivers, France). Injections (volume 100 µL) were performed twice a week with 2 mg/kg of GST, GST-MUC4_EGF1_, and GST-MUC4_EGF2_ domains (wild-type) or the mutated forms GST-EGF1#1 and GST-EGF2#i. Tumor development was evaluated twice a week by measuring the tumor volume to enlighten the tumorigenicity of each domain. * Statistically significant difference between GST-MUC4_EGF1_ and GST; ^#^ statistically significant difference between GST-MUC4_EGF2_ and GST; ^¤^ statistically significant difference between GST-EGF1#1 and GST; ^§^ statistically significant difference between GST-EGF2#i and GST; ^#, o^ *p* < 0.05, **^, ##, oo^ *p* < 0.01, and ***^, ###, ooo, §§§^ *p* < 0.001.

The in vivo tumorigenic potential of MUC4_EGF1_ and MUC4_EGF2_ domains was then studied using a subcutaneous xenograft model of pancreatic cancer. Unfortunately, the amount of purified GST-MUC4_EGF1+2_ was not sufficient to be considered in this assay. The results indicate, once again, that MUC4_EGF1_ had a higher impact than MUC4_EGF2_ on tumor progression in both xenograft models (Figure 5). The effect was significant for Panc-1 xenografts (Figure 5A) and equivalent to hEGF (Supplementary Materials Figure S9). For MiaPaCa-2 xenografts, we could not reach significance as tumors were very aggressive and we had to stop the experiment early on. It, however, showed the same tendency (Figure 5B). We also confirmed that mutations of the binding hotspots in MUC4_EGF1_ and MUC4_EGF2_ domains significantly decreased this tumorigenic activity. Tumor sizes, when treated with MUC4_EGF1_ or MUC4_EGF2_, were bigger than the controls (GST) and were reduced when treated with MUC4_EGF_ mutants, confirming the tumorigenic activity of MUC4_EGF_ domains and the involvement of hotspots of interaction in mediating this activity.

### 3.7. MUC4_EGF1+2_ Domains Are Involved in Intracellular Signaling Pathway Activity

**Figure 6:**
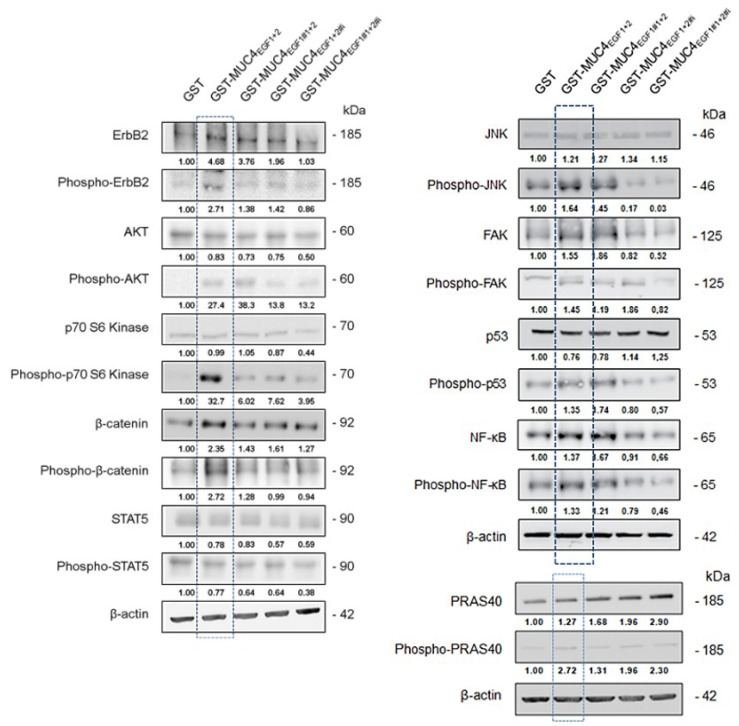
Effects of GST-MUC4_EGF1+2_ and their mutants on intracellular signaling pathway activity. Expression and activation (phosphorylation) of signaling proteins of interest (those activated in phosphokinase arrays, see Figure S12), by Western blotting, in Panc-1 cells treated with GST-MUC4_EGF1+2_, GST- EGF1#1+2, GST- EGF1+2#i, GST- EGF1#1+2#i, or GST alone. Activation levels were obtained after calculating the GST-MUC4/GST ratio normalized to β-actin.

As the MUC4/HER2 complex is known to mediate intracellular signaling, we studied the impact of MUC4_EGF1+2_^,^ as well as of the hotspot mutants (EGF1#1+2, EGF1+2#i, and double mutant EGF1#1+2#i), to identify potential oncogenic targets. Treatment of Panc-1 cells (Supplementary Materials Figure S10A) with GST-MUC4_EGF1+2_ led to the upregulation of specific oncogenic proteins that were similar to those activated by hEGF used as positive control. However, treatment of MiaPaCa-2 cells (Supplementary Materials Figure S10B) with GST-MUC4_EGF1+2_ was less specific, with an upregulation of almost all oncogenic proteins compared with hEGF. Moreover, we confirmed that the mutations of the hotspots of interaction of MUC4_EGF1+2_ led to a strong decrease in these upregulations. Interestingly, some oncogenic proteins extended their upregulation when one of the two domains was mutated, again suggesting different roles and impacts in the signaling pathways triggered by each of the MUC4_EGF_ domains. In Panc-1 cells, out of 84 proteins, 43 were specifically activated by MUC4_EGF1+2_, and most of them were involved in proliferation (29%) and migration (33%). Interestingly, some were involved in survival (24%) and other mechanisms (14%), including angiogenesis (3%) (Supplementary Materials Figures S10B and S11), which highlights the possibility of combined therapies targeting these processes. We then looked at signaling pathways involving activation by phosphorylation (Supplementary Materials Figure S12). We found that MUC4_EGF1+2_ especially activated the p70S6K/AKT/mTOR axis involved in proliferation and the β-catenin pathway involved in both proliferation and migration processes (Supplementary Materials Table S3). Cell treatment with MUC4_EGF1+2_ also generated an activation loop of the Src and Ras pathways with the mobilization of p53 and FAK signaling (promotion of migration), as well as of the ERK and JNK pathways, both involved in proliferation. This effect was almost abrogated in both cell lines when we used MUC4_EGF1+2_ mutants on the hotspots of interaction (Supplementary Materials Figure S12). We confirmed these data by Western blotting for the main activated pathways, with activation (phosphorylation) of HER2, Akt, p70S6 kinase, JNK, FAK, p53, NF-κB, PRAS40, and β-catenin (Figure 6). Interestingly, some proteins such as akt, JNK, p53, and NF-κB remained phosphorylated with EGF1#1+2 mutant while their expression decreased when the ionic cluster or both clusters of MUC4_EGF1+2_ were mutated. These results reinforce the hypothesis of different roles and impacts on the signaling pathways for each of the MUC4_EGF_ domains and concerted and synergistic effects of both combined domains as the ErbB family of receptor-binding partners.

## 4. Discussion

MUC4/HER2 is a pro-oncogenic complex involved in cancer cell tumorigenic properties and in activation of different oncogenic signaling pathways leading to tumor progression in many epithelial cancers (lung, esophagus, colon, breast, and pancreas) [1,3,9,10]. We are particularly interested in finding different strategies to stop or decrease HER2 signaling because HER2 targeting is not efficient in pancreatic cancer [17], and MUC4, which is not expressed on normal healthy pancreas, is neoexpressed very early during pancreatic carcinogenesis in preneoplastic stages [9]. Targeting the MUC4/HER2 complex has, thus, become a promising alternative route to target HER2 driven cancers.

However, nothing is known at the molecular level about the way in which MUC4 and HER2 interact despite the fact that PPI networks play important roles in cellular function and biological processes such as cancer [44]. Moreover, MUC4 presents many drawbacks, as a huge transmembrane protein, highly glycosylated, and with numerous disulfide bridges, making the characterization of its structure very difficult. To overcome these difficulties, we started to decipher the complex at the molecular level for a better understanding of the interaction mechanisms between MUC4 and HER2. We, thus, previously demonstrated that the interaction was located in the EGF domains containing part of MUC4β extracellular domain [10] and, more recently, we quantified the first binding affinity values between MUC4β and HER2 [25], paving the way to the present work.

We first dissected the complex at the molecular level by analyzing deletion mutants of EGF domains of MUC4, as well as single or combined MUC4_EGF_ domains, using an integrative approach, considering in parallel the biophysical interaction and the cellular biological activity. MUC4_EGF1_ appeared as the domain most involved in both binding affinity and cancer cell biological properties, while MUC4_EGF2_ looked secondary for the binding but almost of equal importance for the cellular properties. These results suggest that MUC4_EGF1_ and MUC4_EGF2_ can be considered soluble HER2 ligands with their own binding and biological properties, and that the MUC4_EGF1+2_ combination resumes most of the binding potency and biological activity of MUC4β for HER2. MUC4/HER2 PPI is complex and acts at different levels since, in addition to the direct interaction that we characterized, it was also previously shown that MUC4 regulates the localization of HER2 from intracellular compartments to the plasma membrane [45]. To gain further insight into the molecular basis of the MUC4/HER2 complex and investigate the role of individual amino acids at the interface, the structure of the target protein must be available with a certain level of accuracy as achieved by X-ray crystallography. In the absence of an experimentally established crystal structure, homology modeling is the most accurate technique for 3D structure prediction of proteins [46]. As HER2 has no known ligand and shows an open conformation similar to that of EGFR bound with EGF [47], we chose the crystallized hEGF/EGFR complex (PDB code: 1IVO, [40]) as a high-sequence-homology template in the process of MUC4_EGFs_/HER2 homology modeling. We then considered that each MUC4_EGF_ domain was able to bind HER2 at the same binding site according to in vitro GST-pull down and MST experiments with single GST- MUC4_EGF_ fusion proteins. Reinforcing our approach was the fact that the same methodology was previously used for NRG-1β/ErbB3 and NRG-1β/ErbB4 structures with the crystal structure of hEGF/EGFR complex as a template [48]. The MD simulations led to the identification for the first time of critical aa of MUC4_EGF1_ and MUC4_EGF2_ domains involved in the interaction with HER2. The strength of our data also comes from the total validation of MD simulation data by in vitro studies for both the binding affinity and the biological activity, pointing toward the same important aa. Moreover, the clusters which contain charged aa residues are a critical determinant for receptor binding and suggest a similar mechanism to the one described for R41 of hEGF in the hEGF/EGFR complex [49]. The mutation of these binding hotspots on the MUC4_EGF1+2_ module clearly showed that MUC4_EGF1_ is the domain most involved in the binding affinity. However, both domains are equally potent and strongly mediate the in vitro cancer cell properties. An in vivo experiment further demonstrated the potency of the single MUC4_EGFs_ domains, displaying “growth factor-like” biological activities, as well as the relevance of the identified binding hotspots.

**Figure 7:**
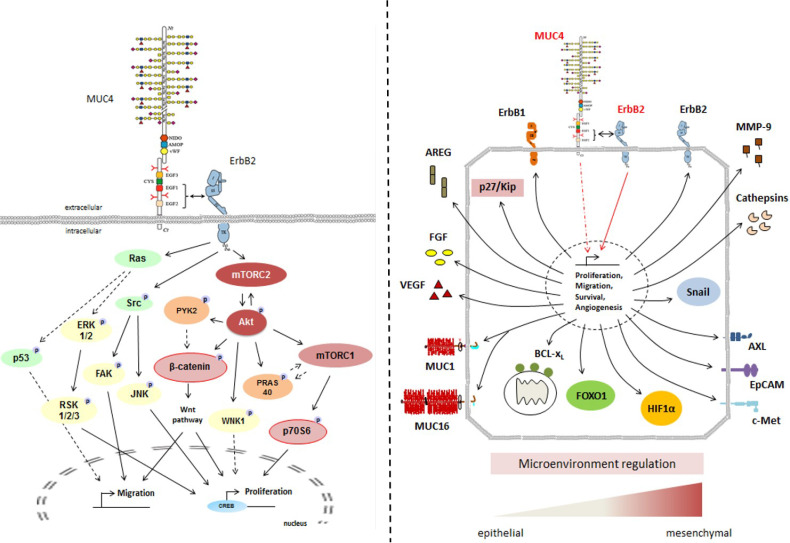
General schemes of MUC4/HER2 interaction at the membrane and subsequent activation of intracellular signaling pathways involved in cell proliferation and migration (**left panel**) and in oncogenic pathways (**right panel**).

The importance of the MUC4_EGF1+2_ domain in mediating activation of downstream oncogenic signaling pathways was also shown, confirming that the combined EGFs module resumed most of the downstream activation of MUC4 with several signaling proteins of interest (mTOR pathway, Akt, and β-catenin). Interestingly, we also found that MUC4_EGF1+2_ regulates the expression of other membrane-bound mucins such as MUC1 and MUC16, two membrane-bound mucins that we recently described as being a potent molecular signature for bad prognosis in pancreatic cancer [50] and overall survival [51]. Moreover, we found many oncogenic actors (nectin, mesothelin, E-selectin, etc.) known to interact with HER2 or MUC16 [52,53], suggesting that they could also interact with MUC4. Interestingly, MUC4_EGF2_ seemed to specifically activate several major pathways since the mutation of the ionic cluster of the MUC4_EGF1+2_ module abolished the phosphorylation of many target kinases (Akt, JNK, and p53), while it was not observed for the EGF1#1+2 mutant. These data support the idea that the MUC4_EGF1+2_ domain represents the minimal sequence of MUC4 to interact with HER2, and that both domains provide concerted and synergistic effects, with MUC4_EGF1_ more involved in the binding activity and MUC4_EGF2_ more involved in the oncogenic signaling pathways. Regarding MUC4 and MUC4/HER2 activation of downstream signaling pathways and their role in the biology of cancer, our studies highlight the importance of the regulation of key tumorigenic processes [9,10] such as proliferation and migration, cell survival, and interaction with the micro-environment [4] (Figure 7). As other membrane mucins (MUC3/MUC17, MUC13, MUC16) possess EGF domains [8,23] and have been shown to interact with ErbB receptors [54,55]), it will be interesting to enlarge and test this PPI approach using these other oncogenic complexes.

## 5. Conclusions

In conclusion, our structure–function relationship study led to a better understanding of the mechanisms involved in the MUC4/HER2 complex formation at the cell surface, linking them to activation of intracellular signaling pathways. These results confirm that the MUC4_EGF1+2_ module represents the minimal sequence of MUC4β to target in order to design small PPI inhibitors with the long-term goal of developing new anticancer drugs targeting the MUC4/HER2 complex. This approach could be extended to other membrane-bound mucins, overexpressed in epithelial cancers, containing EGF domains, and interacting with ErbBs receptors.

## Data Availability

The data presented in this study are available on request from the corresponding authors.

## Supplementary Materials

The following are available online at https://www.mdpi.com/article/10.3390/cancers13225746/s1: Table S1. Sequences of primers used for cloning and site-directed mutagenesis in MUC4_EGF1_ and MUC4_EGF2_; Table S2. List, commercial reference, and dilution of antibodies used for Western blotting, GST pull-down, PLA assay, and coimmunoprecipitation studies; Table S3. Activated proteins following cell treatment with wild-type MUC4_EGF1+2_ and hotspot mutants EGF1#1+2, EGF1+2#i, and EGF1#1+2#i; Figure S1. MUC4 physically interacts with HER2 in vitro and in cellulo; Figure S2. eGFP-MUC4 fusion proteins with EGF1 and EGF2 deletion domain mutants; Figure S3. Impact of MUC4_EGF_ domains on binding affinity; Figure S4. Modeling by homology: construction of the model; Figure S5. Modeling by homology: molecular dynamics; Figure S6. Percentage of interactions involving various (A) EGF1 or (B) EGF2 residues during triplicate MUC4_EGFs_/HER2 MD simulations and clusters identification; Figure S7. Impact of MUC4_EGF_ domains mutations on binding affinity; Figure S8. Positive (hEGF) and negative (PBS) controls in the experiment showing the effect of wild-type MUC4_EGFs_ domains on Panc-1 (A) and MiaPaCa-2 (B) cell proliferation; Figure S9. Positive (hEGF) and negative (PBS) controls in the in vivo experiment showing the effect of MUC4_EGFs_ domains on the tumor progression induced by xenograft of Panc-1 cells (A) or MiaPaCa-2 cells (B); Figure S10. Effects of GST-MUC4_EGF1+2_ on oncogenic pathway activity in Panc-1 and MiaPaca-2 cells; Figure S11. Analysis of the regulated oncogenic proteins by GST-MUC4_EGF1+2_ in Panc-1 and MiaPaCa-2 cells; Figure S12. Activation of signaling pathways by GST-MUC4_EGF1+2_ domain in Panc-1 and MiaPaca-2 cells; Figure S13. Original uncropped western blot.
