# Supplementary material for: The EGF Domains of MUC4 Oncomucin Mediate HER2 Binding Affinity and Promote Pancreatic Cancer Cell Tumorigenesis
Source: Cancers (Basel). 2021 Nov 16;13(22):5746. doi: 10.3390/cancers13225746 (PMC8616066; doi:10.3390/cancers13225746)
Supplement: Supplementary file 1 [file cancers-13-05746-s001.zip › cancers-1431608-supplementary.pdf]

Supplementary Material: The EGF Domains of MUC4 Oncomucin Mediate HER2 Binding Affinity and Promote Pancreatic Cancer Cell Tumorigenesis

Nicolas Stoup, Maxime Liberelle, Céline Schulz, Sumeyye Cavdarli, Romain Vasseur, Romain Magnez, Fatima Lahdaoui, Nicolas Skrypek, Fabien Peretti, Frédéric Frénois, Xavier Thuru, Patricia Melnyk, Nicolas Renault, Nicolas Jonckheere, Nicolas Lebègue and Isabelle Van Seuningen

Table S1. Sequences of primers used for cloning and site-directed mutagenesis in MUC4$_{EGF1}$ and MUC4$_{EGF2}$.

**MUC4$_{EGF}$ domain**	**Forward sequences 5′- 3′**	**Reverse sequences 5′- 3′**
Cloning		
MUC4$_{EGF1}$	CGCGGATCCCAGAACCAGTCCTGC	TCCCCCGGGTCAGAAGCAGCGGCTGTC
MUC4$_{EGF2}$	CGCGGATCCGTGTCCCCGTGCAGT	TCCCCCGGGTCATCTACAGTGCTCGCC
Site-directed Mutagenesis		
**MUC4$_{EGF1}$ cluster 1 mutations**		
mut R38A	CCCCCCAGCCTTCACTGACAGC**GC**CTGCTTC	GAAGCAG**GC**GCTGTCAGTGAAGGCTGGGGGG
mut D36A-F40A	CCAGCCTTCACTG**C**CCAGC**GC**CTGC**GC**CTGACCCG	CGGGTCAG**GC**GCAG**GC**GCTG**G**CAGTGAAGGCTGG
mut Y11A	CCCTGTGAATTACTGC**GC**CAACCAAGGCCACTGCTAC	GTAGCAGTGGCCTTGGTTG**GC**GCAGTAATTCACAGGG
**MUC4$_{EGF1}$ cluster 2 mutations**		
mut T21A-L22A	CACTGCTACATCTCCCAG**G**CT**GC**GGGCTGTCAGC	GCTGACAGCCC**GC**AG**C**CTGGGAGATGTAGCAGTG
mut Q25A-M27A	T**GC**GGGCTGT**GC**GCCC**GC**GTGCACCTGCCC	GGGCAGGTGCAC**GC**GGGC**GC**ACAGCCC**GC**A
**MUC4$_{EGF2}$ ionic cluster mutations**		
mut E40A	GGGGCGAGCACTGTG**C**GTGACCCGGGTCGACTCG	CGAGTCGACCCGGGTCAC**G**CACAGTGCTCGCCCC
mut R6A	CCGTGTCCCCGTGCAGT**GC**GGGCTACTGTGACCATG	CATGGTCACAGTAGCCC**GC**ACTGCACGGGGACACGG
mut E37A	CGGCCTGGGGCG**C**GCACTGTG**C**GTGACC	GGTCAC**G**CACAGTGC**G**CGCCCCAGGCCG

Table S2. List, commercial reference and dilution of antibodies used for western-blotting, GST pull-down, PLA assay and co-immunoprecipitation studies.

**Antibody name**	**Company name & reference**		**Dilution**
**Co-immmunoprecipation studies**			
**HER2 / HER2 (Ab-1)**	RB-103-P	Lab Vision Neomarker	1/500
**phospho-HER2 (Y1248)**	AF1768	R&D System	1/500
**MUC4 (8G7)**	Sc 53-945	Santa-Cruz Biotechnology	1/200
**HER3/ erbB3 (D22C5)**	12708S	Cell signaling	1/500
**β-actin (AC-15)**	(AC-15) A5441	Sigma	1/5000
**Veriblot for IP Detection Reagent (HRP)**	Ab131366	Abcam	1/5000
**PLA assay**			
**MUC4 (8G7)**	Sc 53-945	Santa-Cruz Biotechnology	1/100
**HER2/Neu (C-18)**	Sc 284	Santa-Cruz Biotechnology	1/100
**GST pull-down studies**			
**HER2/ HER2 (29D8)**	2165S	Cell signaling	1/500
**GST (91G1)**	2625S	Cell signaling	1/500
**Western-Blotting studies**			
**HER2/ HER2 (29D8)**	2165S	Cell signaling	1/500
**phospho-HER2 (Y1248)**	AF1768	R&D System	1/500
**EGFR (D38B1)**	4267S XP	Cell signaling	1/500

**phospho-EGFR (Y1173) (53A5)**	4407S	Cell signaling	1/500
**Src (36D10)**	2109S	Cell signaling	1/500
**phospho-Src family (Y416) (D49G4)**	6943S	Cell signaling	1/500
**JNK (56G8)**	9258S	Cell signaling	1/500
**phospho-JNK (T183/Y185) (81E11)**	4668S	Cell signaling	1/500
**FAK (D2R2E)**	13009S	Cell signaling	1/500
**phospho-FAK (Y397) (D20B1)**	8556S	Cell signaling	1/500
**Akt (C67E7)**	4691S	Cell signaling	1/500
**phospho-Akt (S473) (D9E)**	4060S	Cell signaling	1/500
**mTor (7C10)**	2983S	Cell signaling	1/500
**phospho-mTor (S2448) (D9C2)**	5536S	Cell signaling	1/500
**p70 S6 kinase**	9202S	Cell signaling	1/500
**phospho-p70 S6 kinase (T421/S424)**	9204S	Cell signaling	1/500
**p53**	9282S	Cell signaling	1/500
**phospho-p53 (S15)**	9284S	Cell signaling	1/500
**eNOS (D9A5L)**	32027S	Cell signaling	1/500
**phospho-eNOS (S1177)**	9571S	Cell signaling	1/500
**PRAS40**	2610S	Cell signaling	1/500
**phospho-PRAS40 (S183)**	5936S	Cell signaling	1/500
**STAT5 (D2O6Y)**	94205S	Cell signaling	1/500
**phospho-STAT5 (Y694)**	4322S	Cell signaling	1/500
**β-catenin (D10A8) XP**	8480S	Cell signaling	1/500
**phospho-β-catenin (S675) (D2F1)**	4176S	Cell signaling	1/500
**NF-κB p65 (D14E12)**	8242S	Cell signaling	1/500
**phospho- NF-κB p65 (S536) (93H1)**	3033S	Cell signaling	1/500
**β-actin (AC-15)**	(AC-15) A5441	Sigma	1/5000
**Mouse HRP**	31430	Pierce	1/5000
**Rabbit HRP**	31460	Pierce	1/5000

Table S3. Activated proteins following cell treatment with wild-type EGF1+2 and hotspot mutants EGF1#1+2, EGF1+2#i, and EGF1#1+2#i. Ratios over values obtained with cells treated with GST were calculated (Phospho Kinase array, Proteome Profiler™, R&D System).

	**GST-EGF1+2/ GST**	**GST-EGF1#1+2/ GST**	**GST-EGF1+2#i/ GST**	**GST-EGF1#1+2#i/ GST**
	Panc-1	Panc-1	Panc-1	Panc-1
**Activation (Ratio>1.2)**	p38α			
	ERK1/2	ERK1/2	ERK1/2	ERK1/2
	EGFR			
	MSK1/2			
	AMPKα1			
	AKT1/2/3 (S473)		AKT1/2/3 (S473)	AKT1/2/3 (S473)
	CREB	CREB	CREB	CREB
	HSP27			
	B-catenin			
	p70S6K (T389/T421/S424)			
	p53 (S15)			
	Src			
	Lyn		Lyn	
	Lck		Lck	
	STAT5			

	RSK1/2/3			
	eNOS			
	Fyn			
	Yes			
	Fgr		Fgr	Fgr
	STAT6			
	STAT3 (Y705)			
	p27			
	PLC-γ1			
	Hck			
	Chk-2			
	FAK		FAK	FAK
	PDGFR			
	WNK1		WNK1	WNK1
	PYK2			
**Activation (Ratio>2)**	ERK1/2	ERK1/2	ERK1/2	ERK1/2
	JNK1/2/3	JNK1/2/3		
	GSK-3α/β	GSK-3α/β	GSK-3α/β	GSK-3α/β
	MSK1/2		MSK1/2	MSK1/2
	AMPKα1	AMPKα1		
	AKT1/2/3 (S473)			
	CREB			
	p70S6K (T389/T421/S424)	p70S6K (T421/S424)		
	p53 (S15)	p53 (S15)	p53 (S15)	
	c-Jun		c-Jun	
	Src			
	p27			
	Chk-2			
	FAK			
	STAT3 (S727)			
	WNK1			WNK1
	PYK2			
	PRAS40			PRAS40
		HSP60		

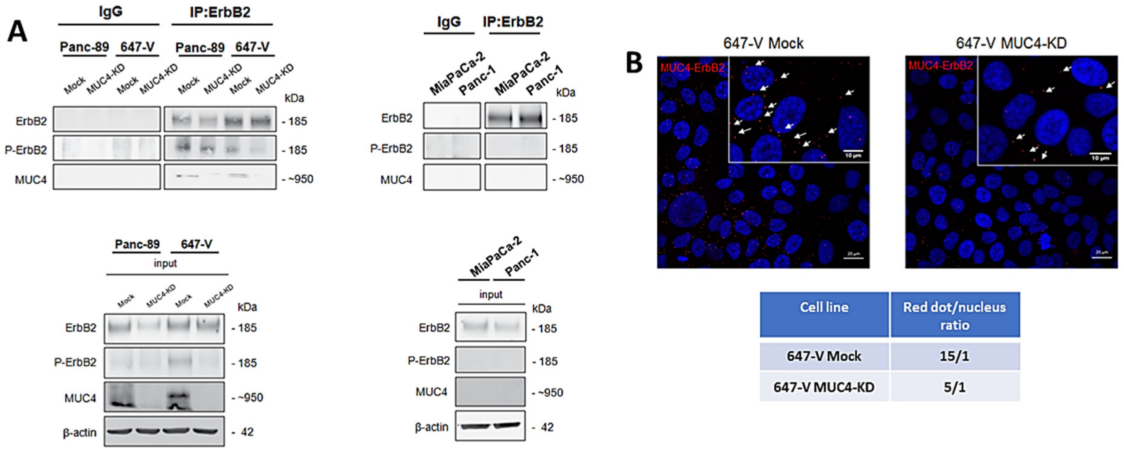

Figure S1. MUC4 physically interacts with HER2 in vitro and in cellulo. (**A**) Co-immunoprecipitation of MUC4 and phospho-HER2 (P-HER2) after immunoprecipitation of HER2 with anti-HER2 antibody in MUC4-expressing (Panc-89 Mock, 647-V Mock) and MUC4 non-expressing (Panc-89 MUC4-KD, 647-V MUC4-KD, MiaPaCa-2 and Panc-1) cancer cell lines. The input shows the relative expression of studied proteins in the respective cell lysate before the immunoprecipitation. (**B**) Proximity Ligation Assay showing co-localisation (white arrows) of MUC4 and HER2 in 647-V Mock and 647-V MUC4-KD cells. Ratios indicate the numbers of MUC4-HER2 interactions (red dots) per nucleus.

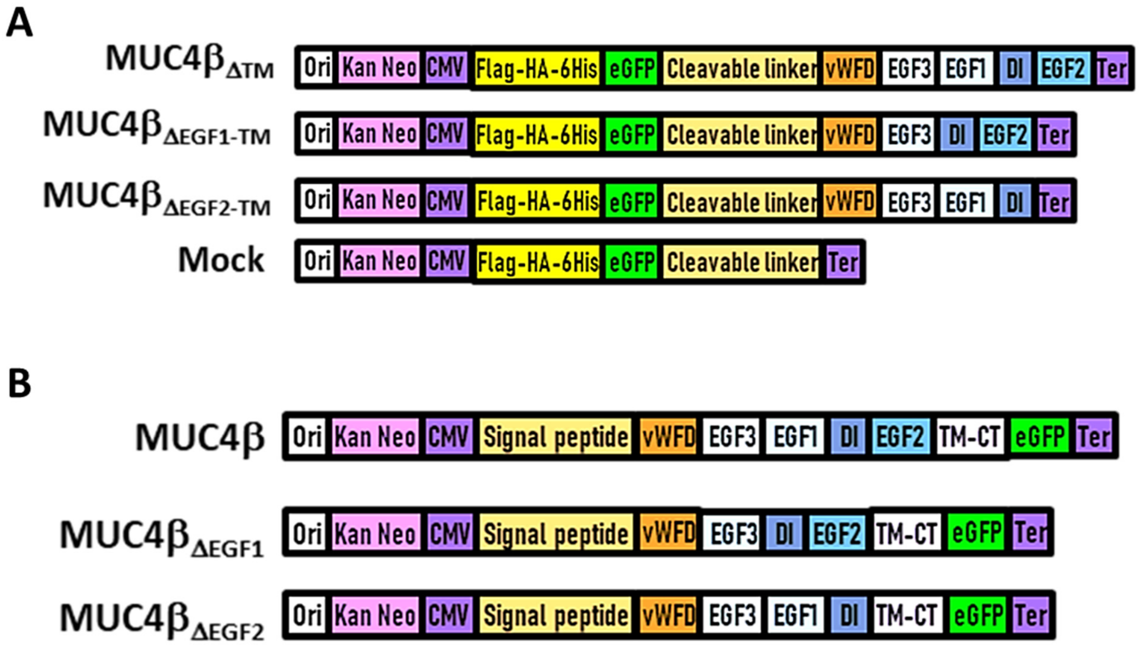

Figure S2. eGFP-MUC4 fusion proteins with EGF1 and EGF2 deletion domain mutants. (**A**) Sequences of eGFP constructs each deleted with one EGF domain of MUC4β. The transmembrane domain (TM) of MUC4β is not present in these constructs. (**B**) The transmembrane domain (TM) of MUC4β and the signal peptide are present in these constructs. Ori: Replication Origin; Kan Neo (Kanamycine, Neomycine): genes for bacteria and eucaryote antibiotic selection; CMV: cytomegalovirus ubiquitous promoter; Flag-HA-6His: triple peptide tag; eGFP: sequence of eGFP fluorescent protein; cleavable linker: cleavage site of TEV protease flanked with 2 peptide spacers (SGS); Signal peptide : original signal sequence of MUC4; vWFD, EGF3, EGF1, DI, EGF2: domains present in MUC4β; TM-CT: TransMembrane domain and Cytosolic Tail; Ter: transcription terminator. Mock is the empty eGFP vector (negative control).

A

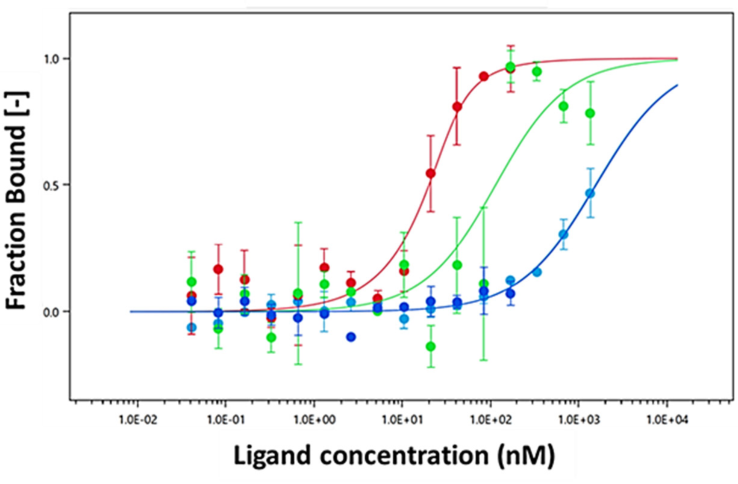

Proteins	Kd
MUC4β$_{ΔTM}$	4.3 ± 3.2 nM
MUC4β$_{ΔEGF2-TM}$	97 ± 75 nM
MUC4β$_{ΔEGF1-TM}$	> 2 000 nM
Mock	> 2 000 nM

B

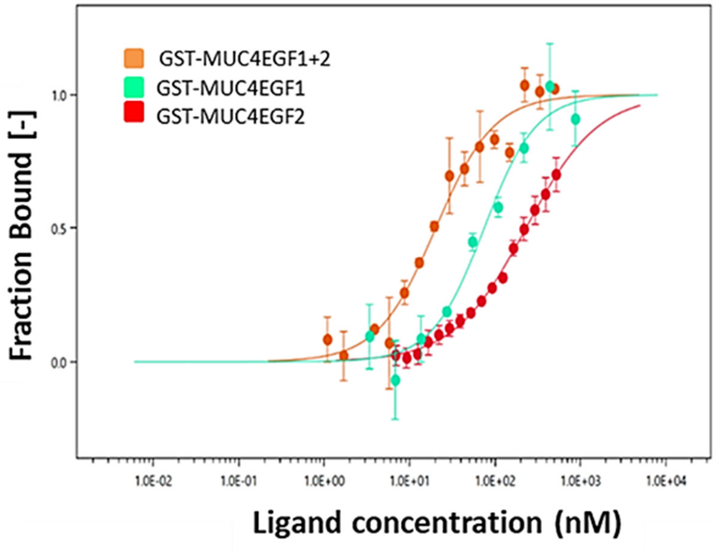

Proteins	Kd
MUC4$_{EGF1+2}$	20 ± 3 nM
MUC4$_{EGF1}$	75 ± 19 nM
MUC4$_{EGF2}$	242 ± 24 nM

Figure S3. Impact of MUC4$_{EGF}$ domains on binding affinity. (**A**): Dose-response binding curves using MST between recombinant HER2 and eGFP-MUC4β$_{ΔTM}$ containing the EGF domains (MUC4β$_{ΔTM}$, red curve, K_d = 4.3 ±3.2 nM) and the deleted forms for each EGF domain: eGFP-MUC4β$_{ΔEGF1}$ (MUC4β$_{ΔEGF1}$, purple curve, K_d > 2000 nM), eGFP-MUC4β$_{ΔEGF2}$ (MUC4β$_{ΔEGF2}$, green curve, K_d = 97 ±75 nM), or eGFP alone (Mock, negative control, blue curve, K_d > 2000 nM), using MST. (**B**) Dose-response binding curves between recombinant fluorescent tagged HER2 and GST-MUC4$_{EGF1+2}$ (MUC4$_{EGF1+2}$, orange curve, K_d = 20 ± 3 nM), GST-MUC4$_{EGF1}$ (MUC4$_{EGF1}$, green curve, K_d = 75 ± 19 nM), and GST-MUC4$_{EGF2}$ (MUC4$_{EGF2}$, red curve, K_d = 242 ± 24 nM).

A

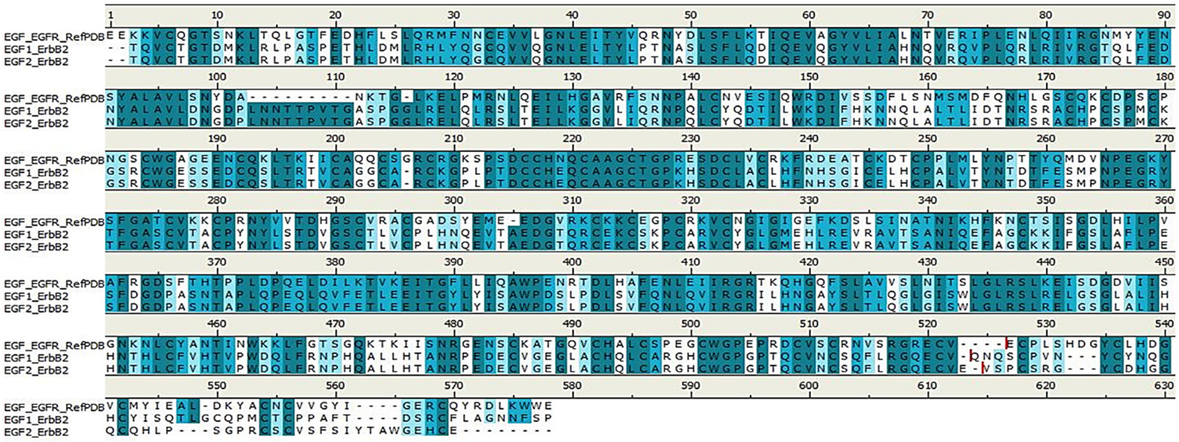

B

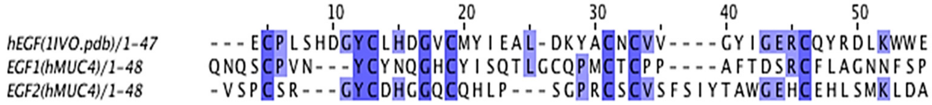

C

	EGFR/HER2	hEGF/MUC4$_{EGF1}$	hEGF/MUC4$_{EGF2}$	MUC4$_{EGF1}$/MUC4$_{EGF2}$	hEGF-EGFR /MUC4$_{EGF1}$-HER2	hEGF-EGFR /MUC4$_{EGF2}$-HER2
Identity	44%	22%	26%	17%	42%	42%
Homology	63%	39%	38%	31%	61%	61%

Figure S4. Modeling by homology: Construction of the model. Multiple sequence alignment between (**A**) human EGF/EGFR of structural crystal template 1IVO.pdb and MUC4$_{EGF1}$ and MUC4$_{EGF2}$/HER2 complexes (EGF1_ErbB2 and EGF2_ErbB2) and (B) hEGF/EGF1/EGF2. (C) Rates of homology and identity sequence.

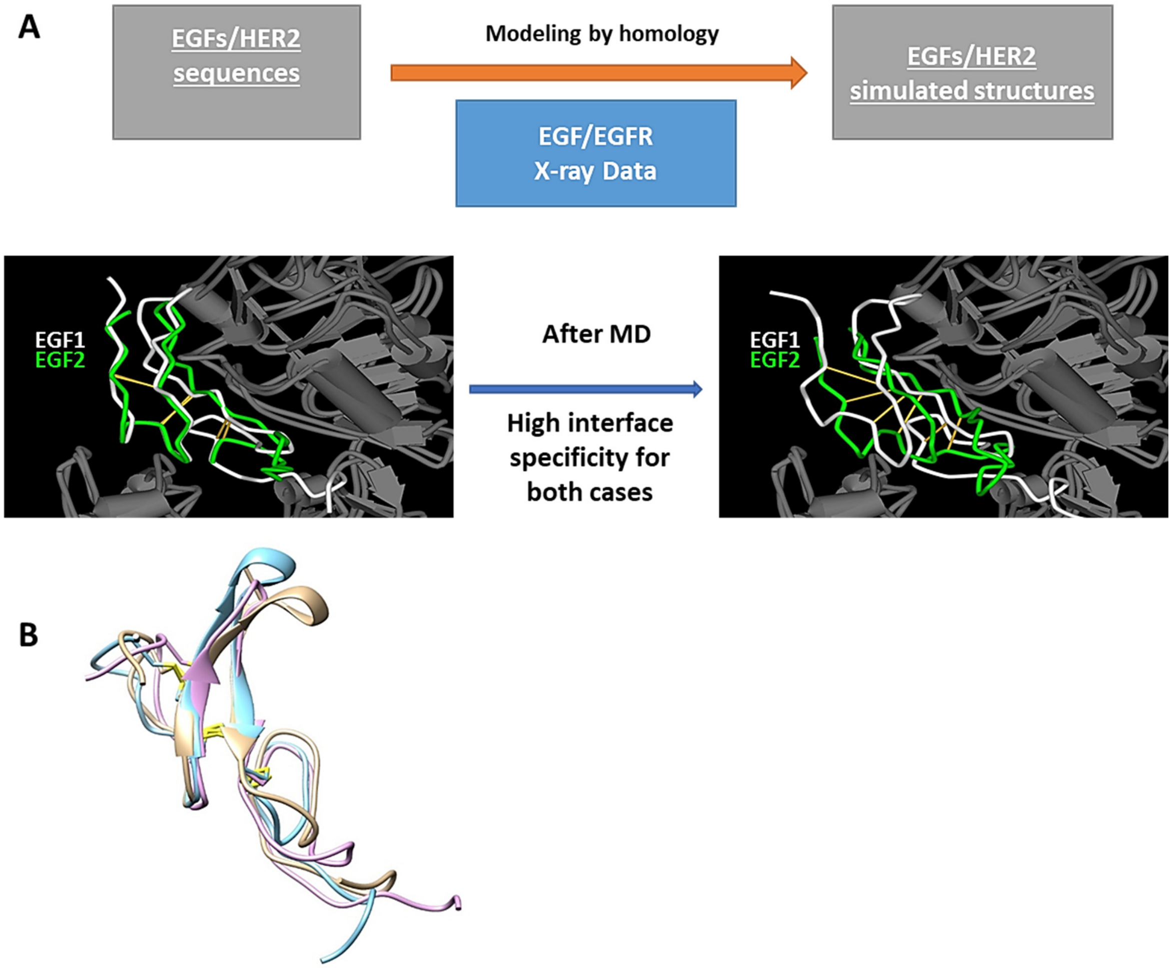

Figure S5. Modeling by homology: Molecular Dynamics. **(A)** MD simulations of $MUC4_{EGFs}$/HER2 sequences from hEGF/EGFR X-ray data leading to $MUC4_{EGFs}$/HER2 simulated 3D structures with high protein interface specificities, **(B)** Structural comparison of the predicted 3D structures of EGF1 (brown) and EGF2 (pink) with the solved 3D structure of the human EGF (blue) available in the Protein Data Bank (PDB) (pdb code: 1jl9). This comparative system indicates that the topology of the $MUC4_{EGF}$ domains are very similar to that of hEGF with standard deviations between the alpha carbons values (rmsd) under 2Å. Illustration was made by UCSF Chimera 1.10.2.

A

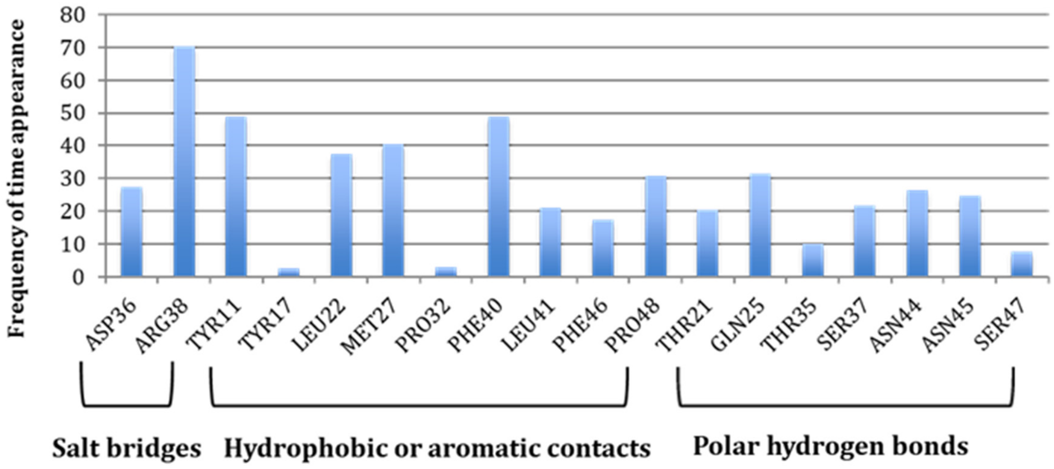

B

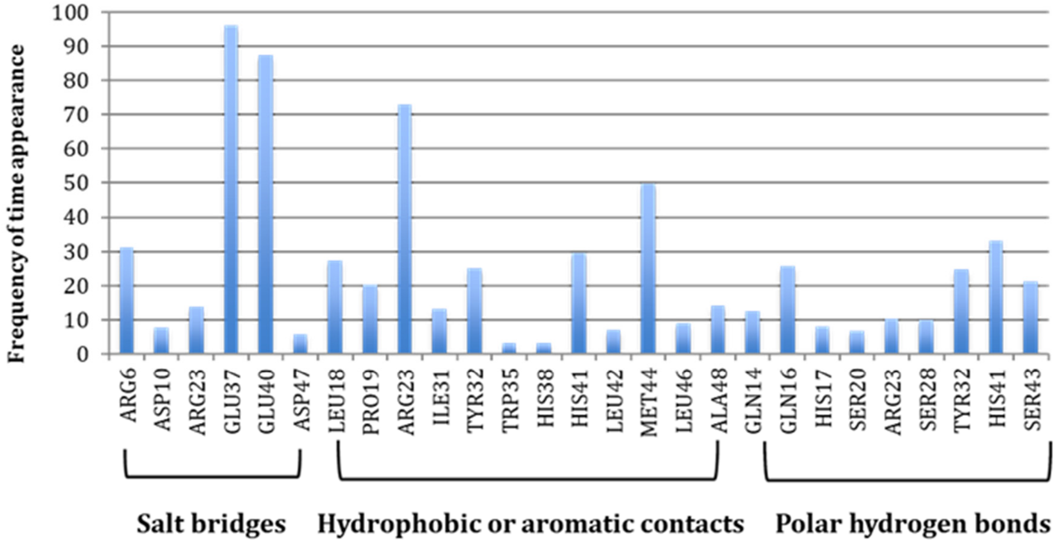

C

	$MUC4_{EGF1}$	HER2	Interaction type
	Y11	R332 (III)	π-cation
	D36	R332 (III)	salt bridge
Cluster 1	R38	E330 (III)	salt bridge
	F40	Y389 (III)	hydrophobic aromatic
	F40	L365 (III)	hydrophobic
	T21	H66 (I)	H-bond
	L22	F86 (I)	hydrophobic
Cluster 2	L22	L95 (I)	hydrophobic
	Q25	E87 (I)	H-bond
	M27	L11 (I)	hydrophobic

	$MUC4_{EGF2}$	HER2	Interaction type
	R6	D362 (III)	salt bridge
	E40	R332 (III)	salt bridge
Cluster 1	H41	H415 (III)	hydrophobic aromatic
	H41	Y389 (III)	hydrophobic aromatic
	M44	L445 (III)	hydrophobic
	R23	H66 (I)	π-cation
Cluster 2	I31	H26 (I)	hydrophobic
	Y32	M23 (I)	hydrophobic
	E37	K10 (I)	salt bridge
	E37	R12 (I)	salt bridge

Figure S6. Percentage of interactions involving various (A) EGF1 or (B) EGF2 residues during triplicate $MUC4_{EGFs}$/HER2 MD simulations and clusters identification. The frequency of aa involved in intermolecular interactions over all triplicate MD simulations was computed according to an average weighted by the time of appearance. Residues are partitioned into three categories of interactions. (C) Clusters of binding residues at the $MUC4_{EGF1}$/HER2 (left table) and $MUC4_{EGF2}$/HER2 (right table) interfaces. The HER2 domain corresponding to each considered aa appears within brackets. Only residue–residue contacts with averaged contact area above 20 Å^2 are displayed. The hydrogen bonds fulfill the criteria that the donor–acceptor distance and angle cutoffs of 3.5 Å and 30° are maintained during at least 50% of the last 50 ns of the trajectory. The maximal distance satisfying salt bridges was set to 4.0 Å.

A

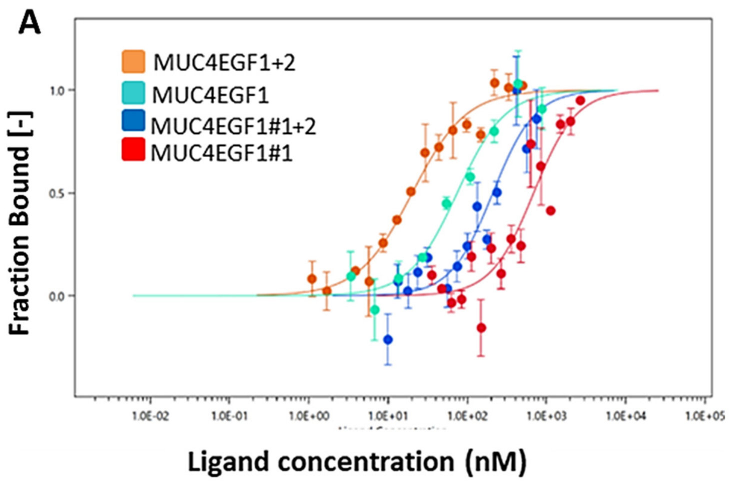

Proteins	Kd
MUC4EGF1+2	20 ± 3 nM
MUC4EGF1	75 ± 19 nM
MUC4EGF1#1+2	233 ± 58 nM
MUC4EGF1#1	629 ± 92 nM

B

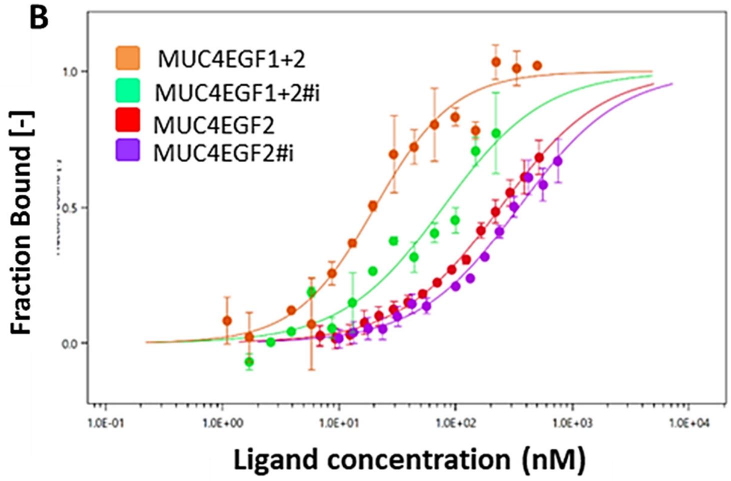

Proteins	Kd
MUC4EGF1+2	20 ± 3 nM
MUC4EGF11+2#i	78 ± 37 nM
MUC4EGF2	242 ± 24 nM
MUC4EGF2#i	346 ± 70 nM

Figure S7. Impact of MUC4$_{EGF}$ domains mutations on binding affinity. **(A)** Dose-response binding curves using MST with fluorescent tagged recombinant HER2 comparing interactions of wild-type GST-MUC4$_{EGF1+2}$ (orange, K_d = 20 ± 3 nM) and GST-MUC4$_{EGF1}$ (cyan, K_d = 75 ± 19 nM) with GST-MUC4$_{EGF1\#1+2}$ (blue, K_d = 233 ± 58 nM) and GST-MUC4$_{EGF1\#1}$ (red, K_d = 629 ± 92 nM) mutants. **(B)** Dose-response binding curves using MST with fluorescent tagged recombinant HER2 comparing interactions of wild-type GST-MUC4$_{EGF1+2}$ (orange, K_d = 20 ± 3 nM) and GST-MUC4$_{EGF2}$ (red, K_d = 242 ± 24 nM) with GST-MUC4$_{EGF1+2\#i}$ (green, K_d = 78 ± 37 nM) and GST-MUC4$_{EGF2\#i}$ (purple, K_d = 346 ± 70 nM) mutants. The double mutant GST-MUC4$_{EGF1\#1+2\#i}$ did not display any binding event (not shown).

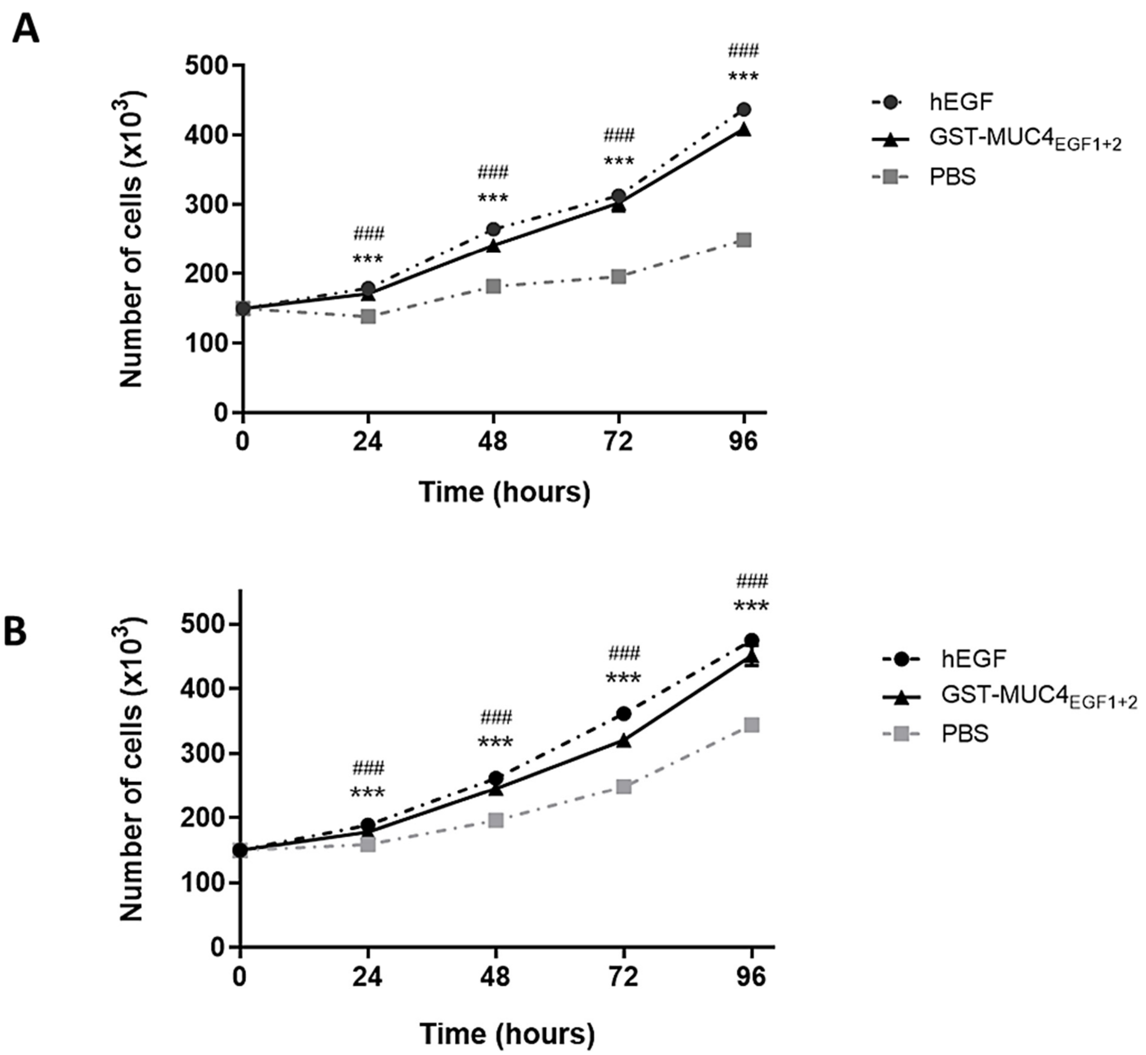

Figure S8. Positive (hEGF) and negative (PBS) controls in the experiment showing the effect of wild-type $MUC4_{EGFs}$ domains on Panc-1 (A) and MiaPaCa-2 (B) cell proliferation. Cells were treated with 25 ng of hEGF (positive control) or 1X PBS (negative control). The curves with GST-$MUC4_{EGF1+2}$ from Fig. 4A (Panc-1) and Fig. 4B (MiaPaCa-2) are reminded.

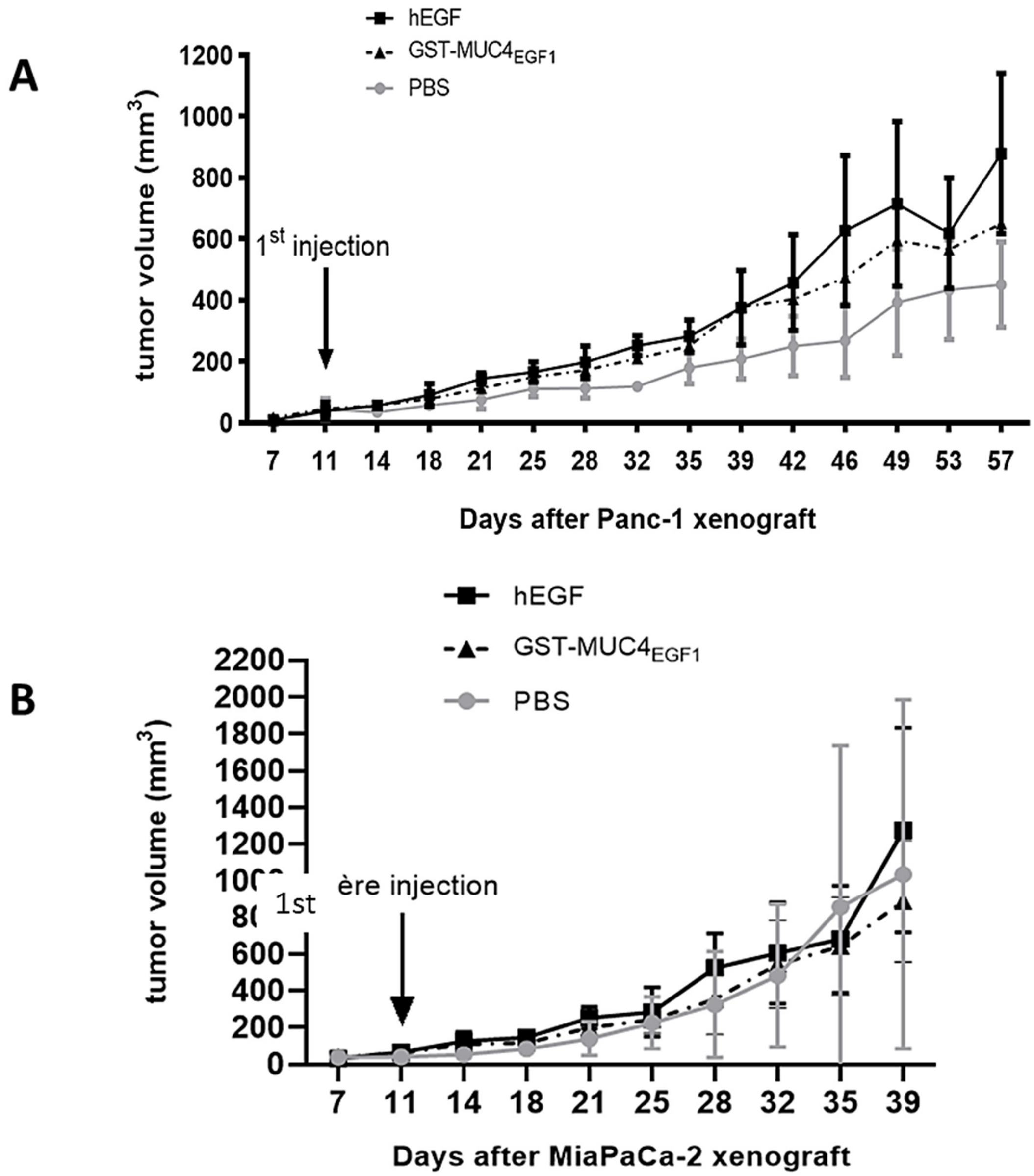

Figure S9. Positive (hEGF) and negative (PBS) controls in the *in vivo* experiment showing the effect of $MUC4_{EGFs}$ domains on the tumor progression induced by xenograft of Panc-1 cells (A) or MiaPaCa-2 cells (B). SCID mice were treated with 50µg/kg (25 ng) of hEGF (positive control) or 2mg/kg (50 µg) of 1X PBS (negative control). The curves with GST-$MUC4_{EGF1}$ from Fig. 5A (Panc-1) and Fig. 5B (MiaPaCa-2) are reminded.

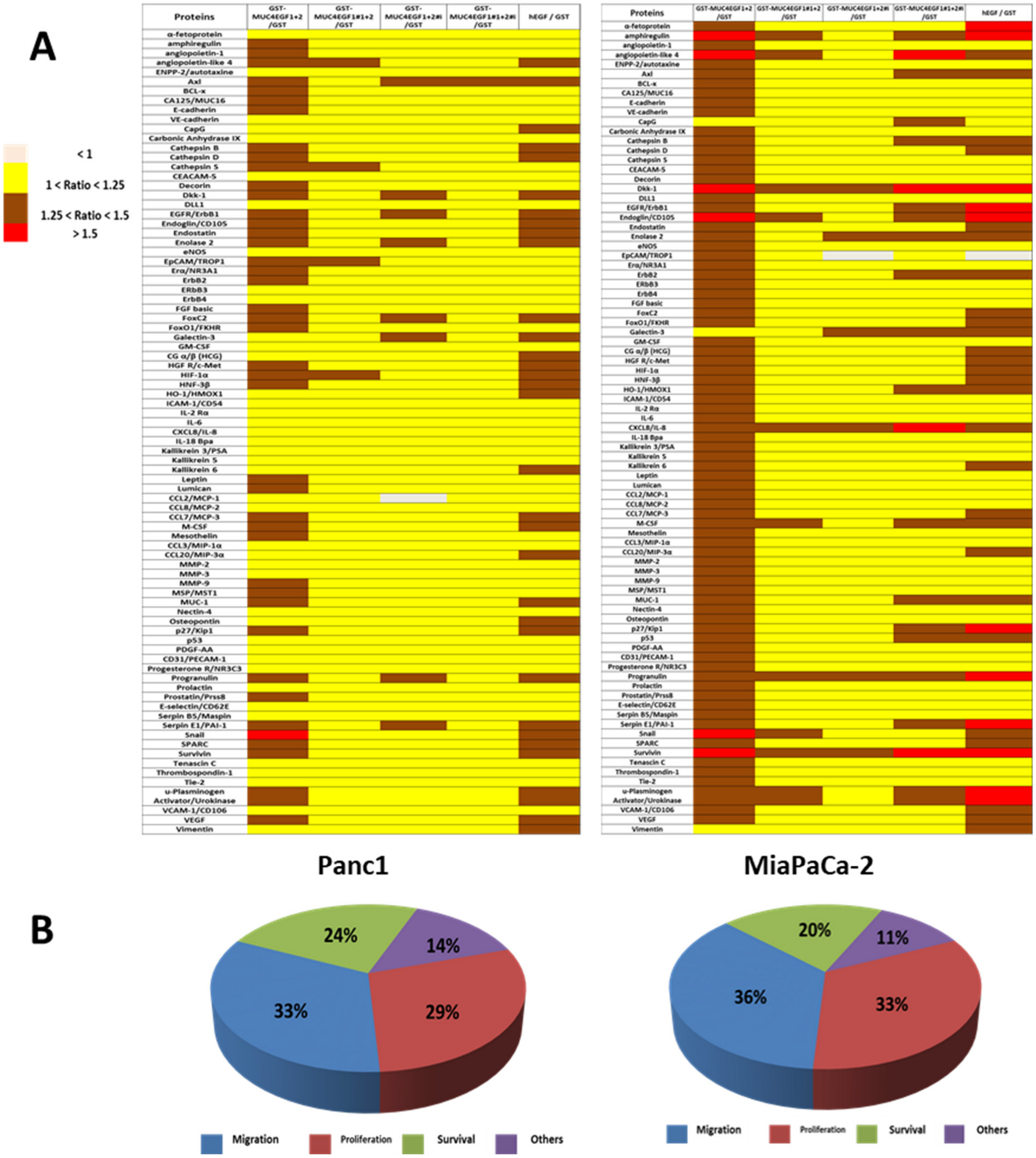

Figure S10. Effects of GST-MUC4EGF1+2 on oncogenic pathway activity in Panc-1 and MiaPaca-2 cells. **(A)** Increased expression of proteins after Panc-1 (left panel) or MiaPaCa-2 (right panel) cell treatment with wild-type GST-EGF1+2, GST-EGF1#1+2, GST-EGF1+2#i and GST-EGF1#+2#i mutants or GST (negative control), and hEGF (positive control) followed by cell extract preparation and protein expression analysis using XL-Onco array (2 experiments, n=4). GST-EGF1+2/GST, GST-EGF1#1+2/GST, GST-EGF1+2#i/GST and GST-EGF1#+2#i/GST ratios were calculated to get the percentage of activation by the EGF domains. Protein activation was ranked as follows: ratio<1.0 (beige); 1<ratio<1.25 (yellow); 1.25<ratio<1.5 (brown) and ratio>1.5 (red). (B) Analysis of and percentage of the proteins activated by type of function (proliferation, migration, survival, others).

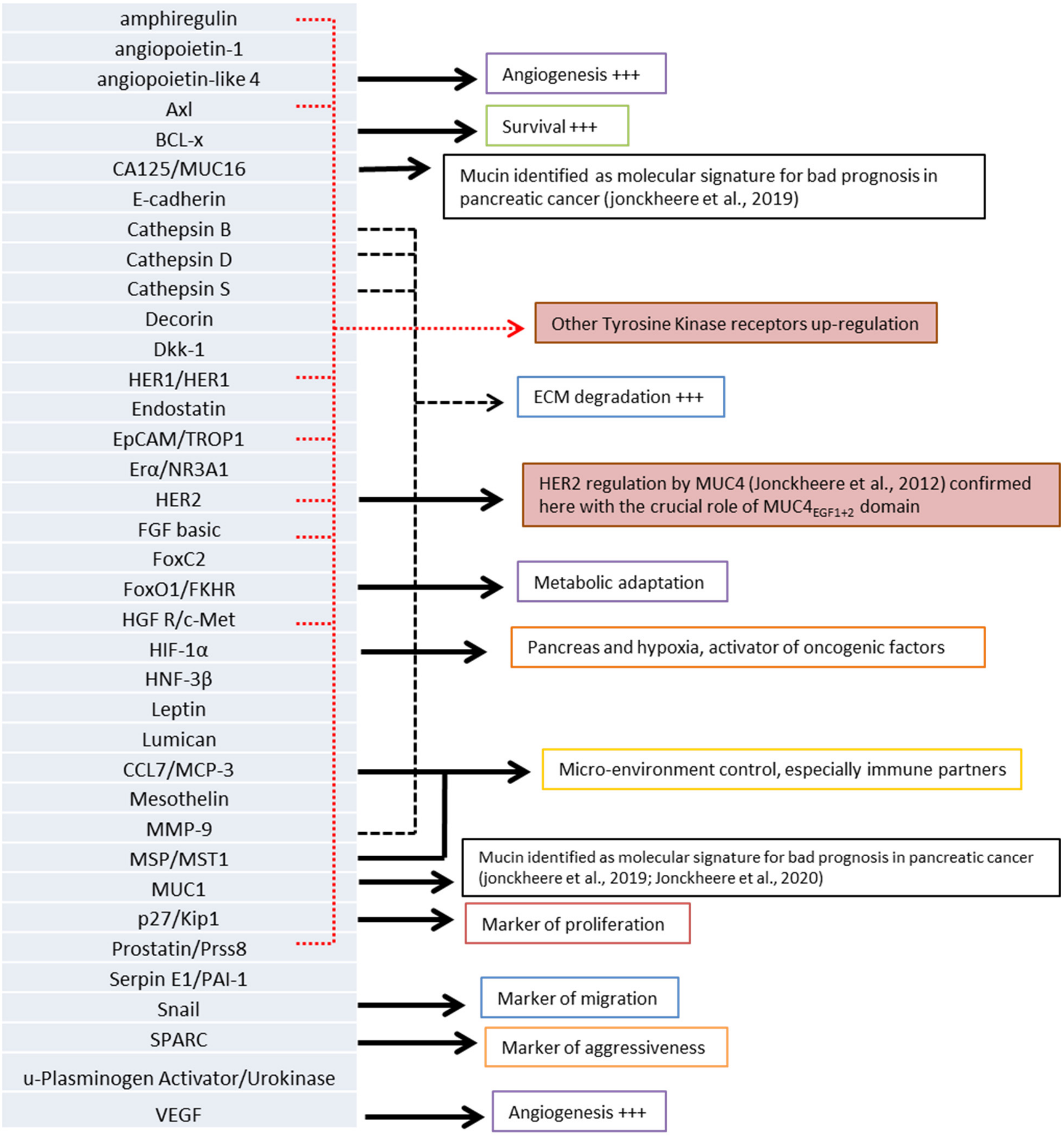

Figure S11. Analysis of the regulated oncogenic proteins by GST-EGF1+2 commonly in Panc-1 and MiaPaCa-2 cells. Data were obtained from analysis of 2 separate experiments using human ONCO-XL array in duplicate (n=4) for each cell line. Results confirm that EGF1+2 regulates major biological axes favoring oncogenic processes such as proliferation and migration.

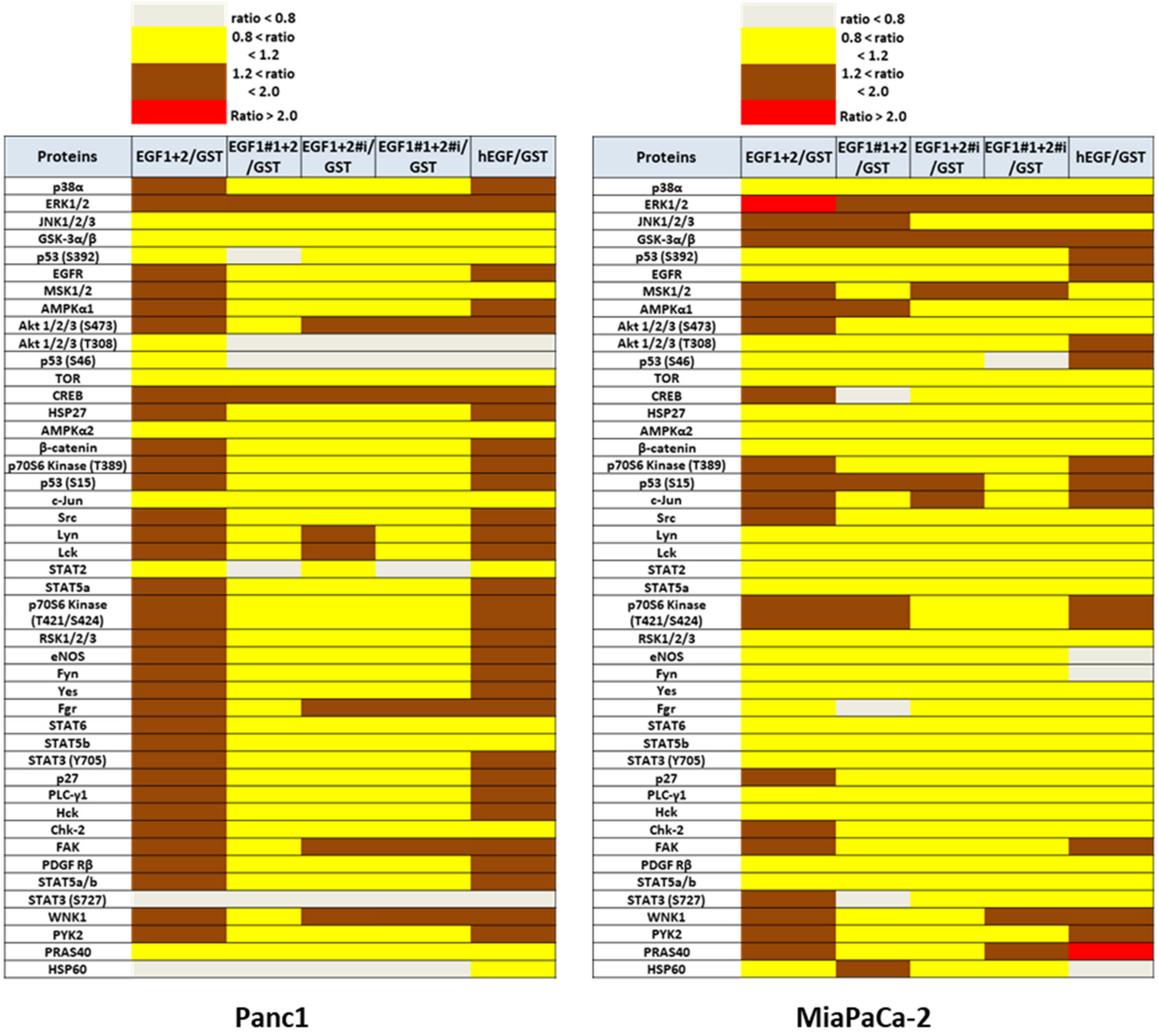

Figure S12. Activation of signaling pathways by GST-EGF1+2 domain in Panc-1 and MiaPaca-2 cells. A human phosphokinase array was used (2 experiments in duplicate, n=4). Determination of spot intensity ratio over GST after treatment of Panc-1 (left panel) and MiaPaca-2 (right panel) cells by GST-EGF1+2, GST-EGF1#1+2, GST-EGF1+2#i, EGF1#1+2#i or GST alone (negative control). hEGF was used as positive control. Kinase activation was ranked as follows: ratio<0.8 (beige); 0.8<ratio<1.20 (yellow); 1.20<ratio<2.0 (brown) and ratio>2.0 (red).

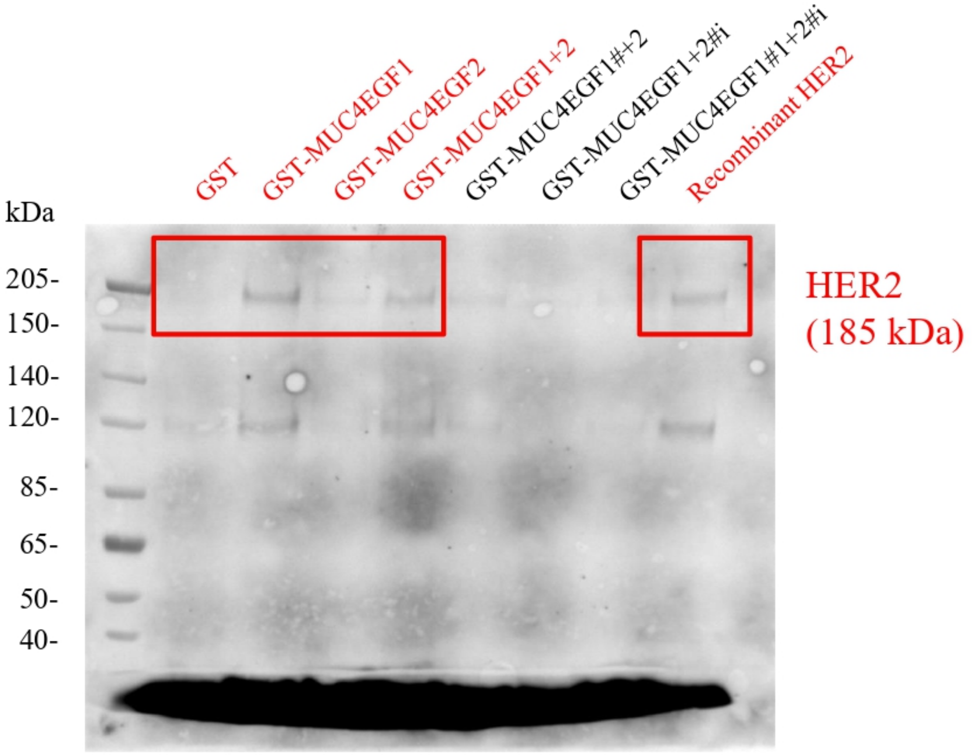

Detail information about Figure 2A. GST pull-down HER2 (185 kDa) uncropped blot.

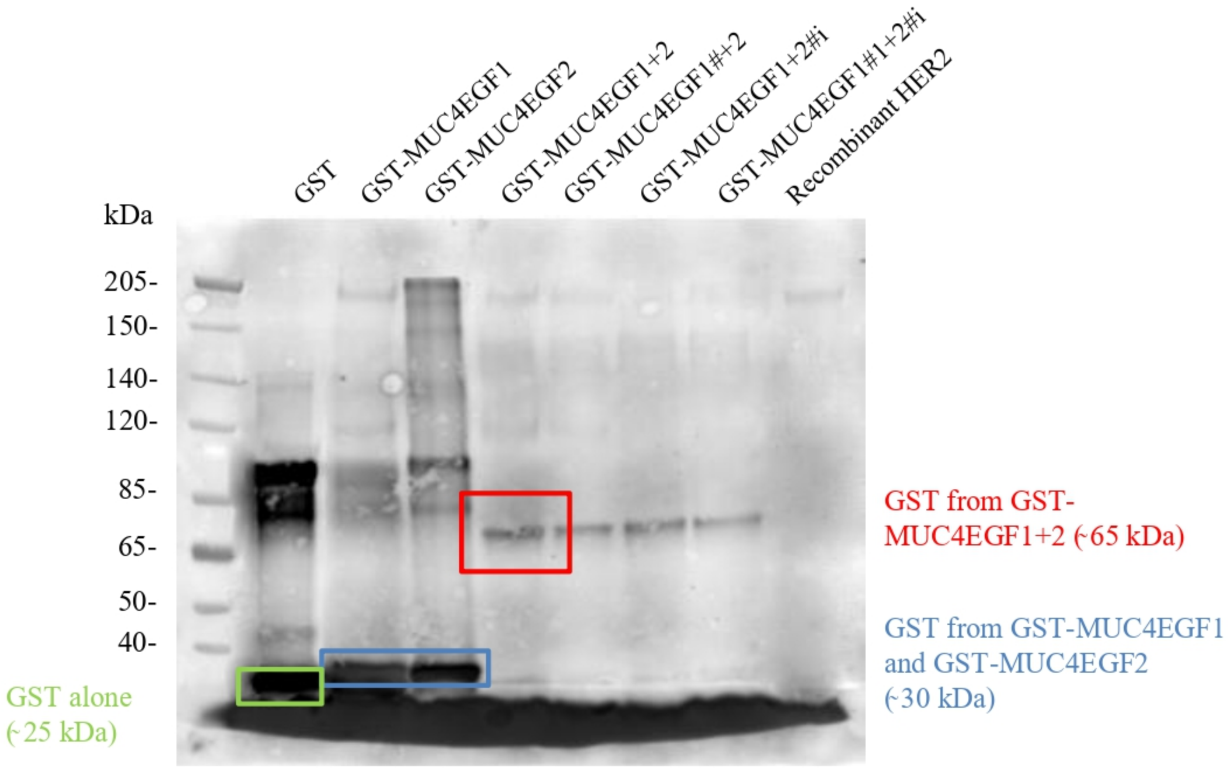

Detail information about Controls Figure 2A. GST pull-down : GST Controls (25-65 kDa) uncropped blot.

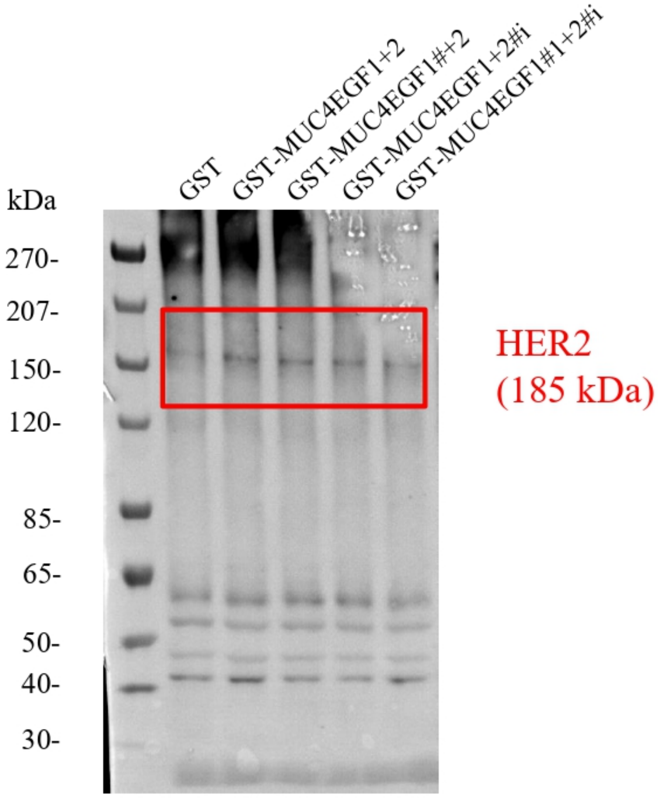

Detail information about Figure 6. HER2 (185 kDa) uncropped blot.

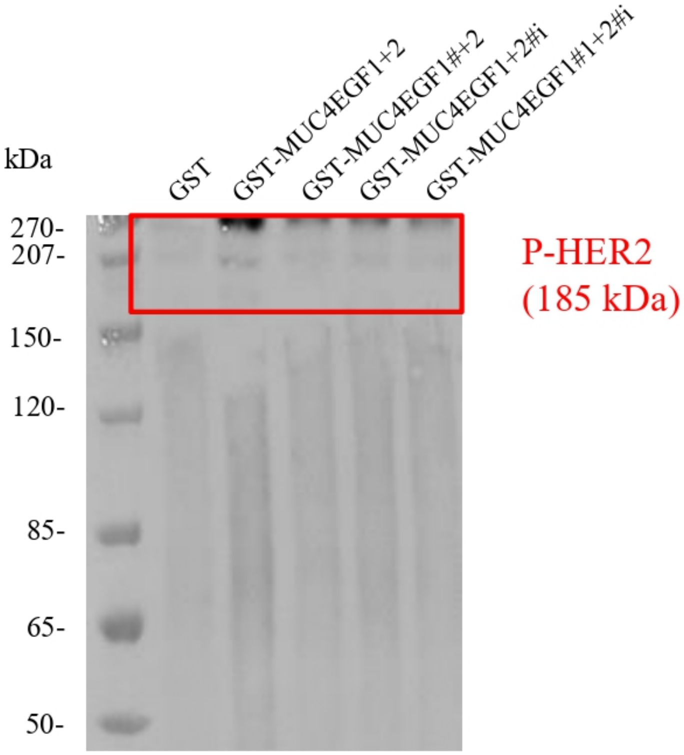

Detail information about Figure 6. Phospho-HER2 (185 kDa) uncropped blot.

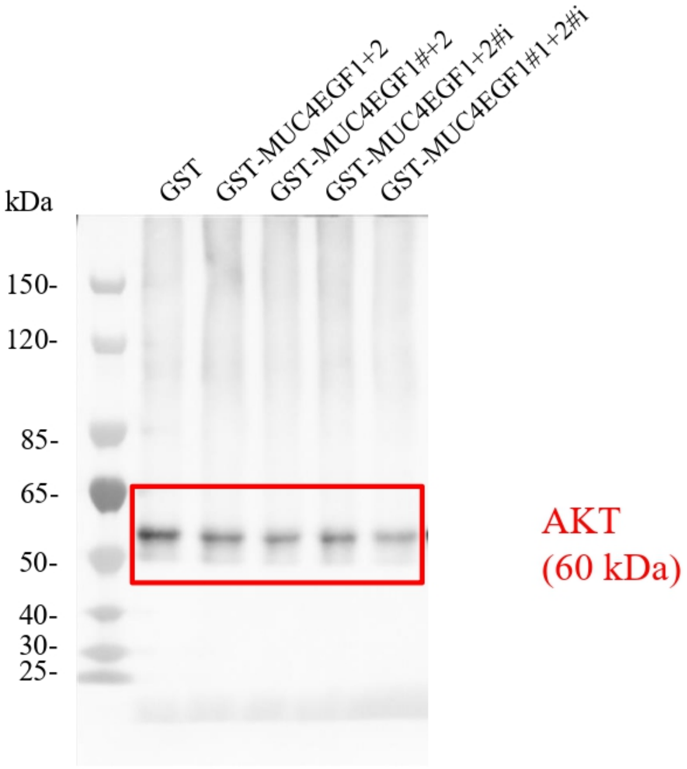

Detail information about Figure 6. AKT (60 kDa) uncropped blot.

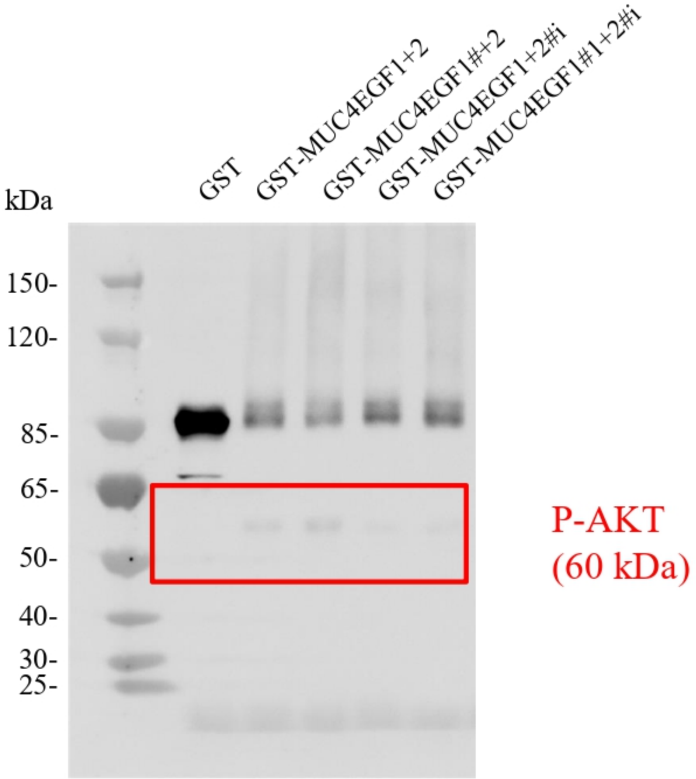

Detail information about Figure 6. Phospho-AKT (60 kDa) uncropped blot.

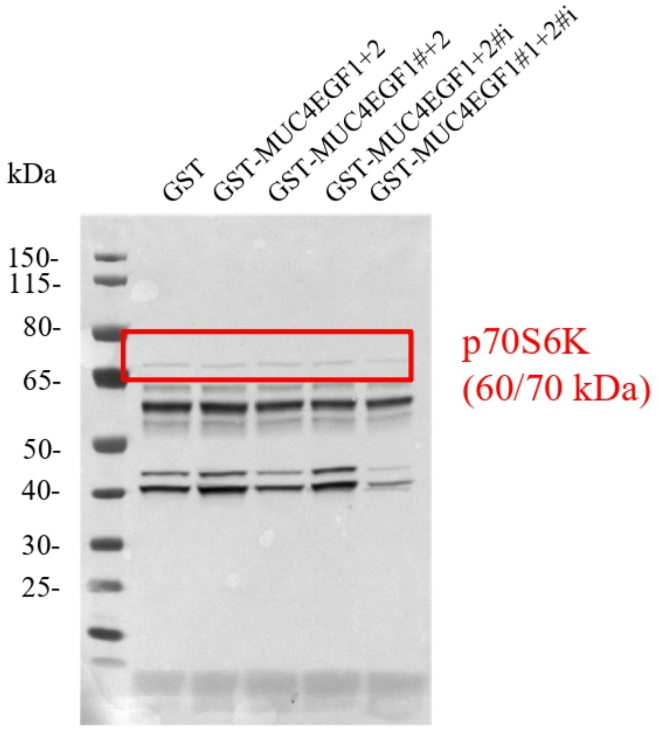

Detail information about Figure 6. p70S6K (60/70 kDa) uncropped blot.

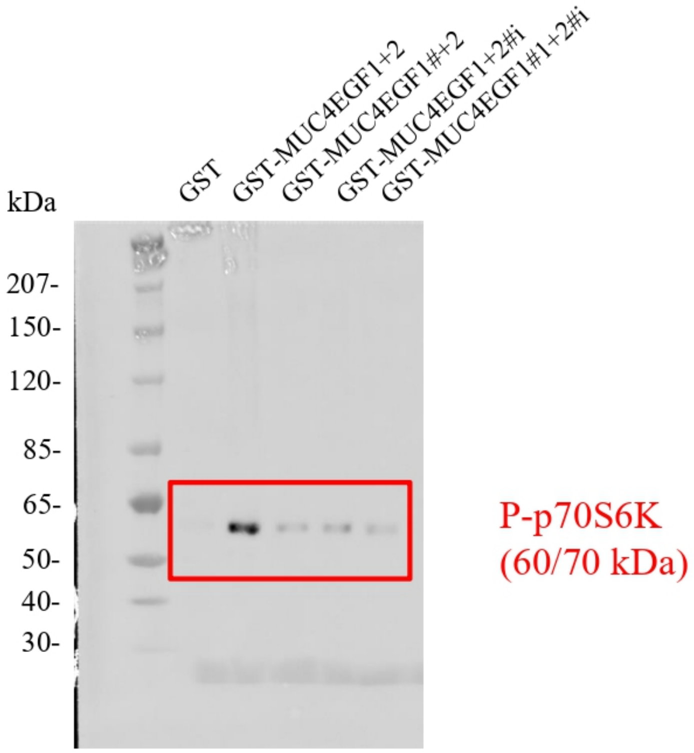

Detail information about Figure 6. Phospho-p70S6K (60/70 kDa) uncropped blot.

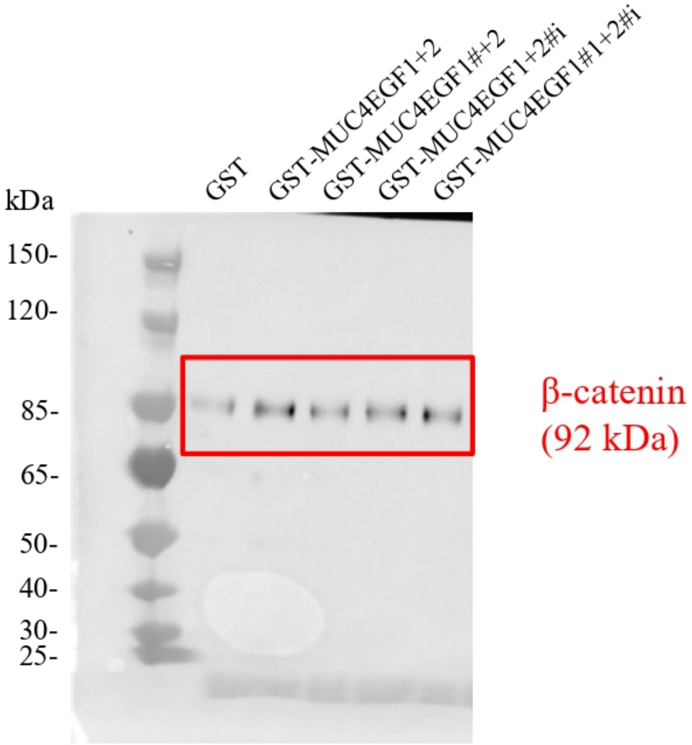

Detail information about Figure 6. β-catenin (92 kDa) uncropped blot.

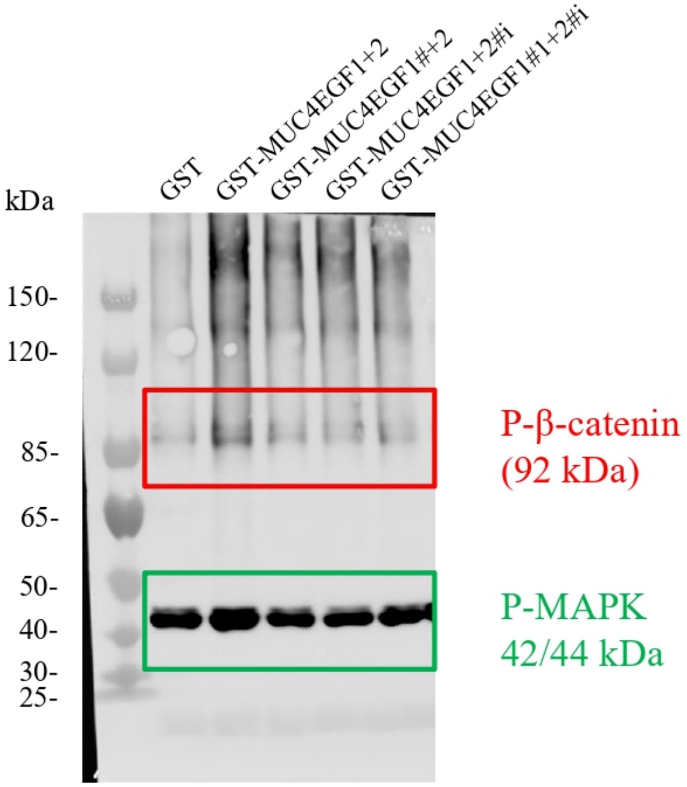

Detail information about Figure 6. Phospho-β-catenin (92 kDa) uncropped blot.
Phospho-MAPK is also present on the membrane (green card, unpublished)

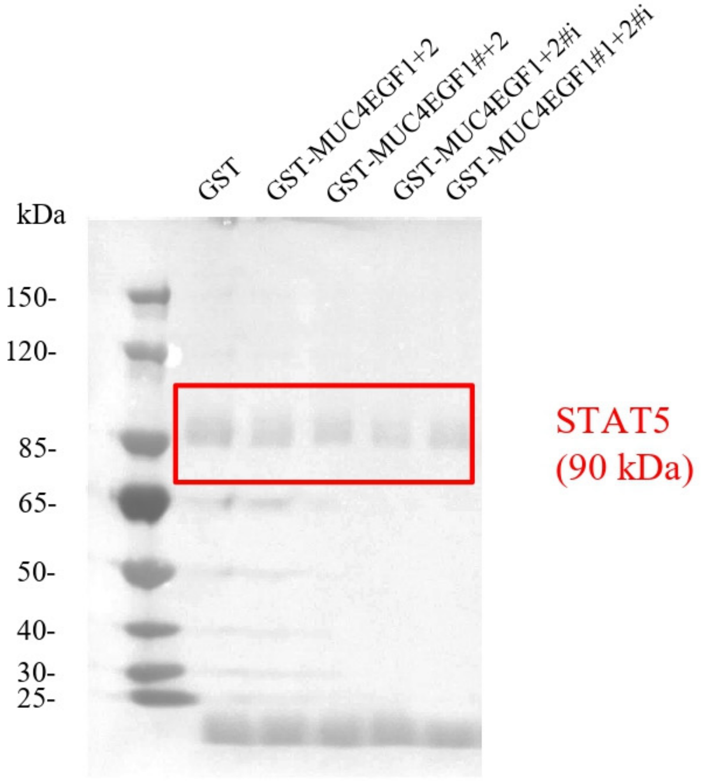

Detail information about Figure 6. STAT5 (90 kDa) uncropped blot.

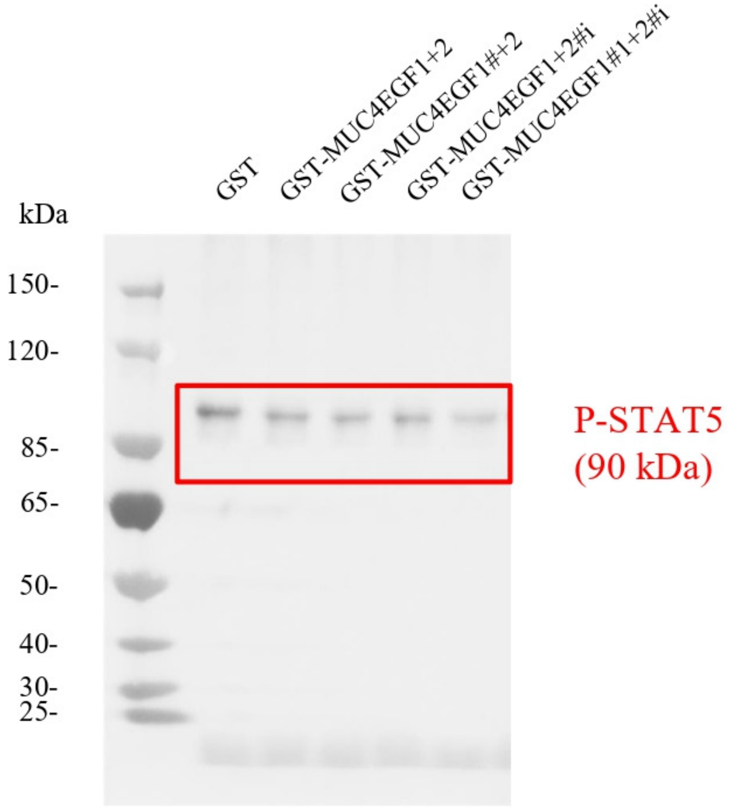

Detail information about Figure 6. Phospho-STAT5 (90 kDa) uncropped blot.

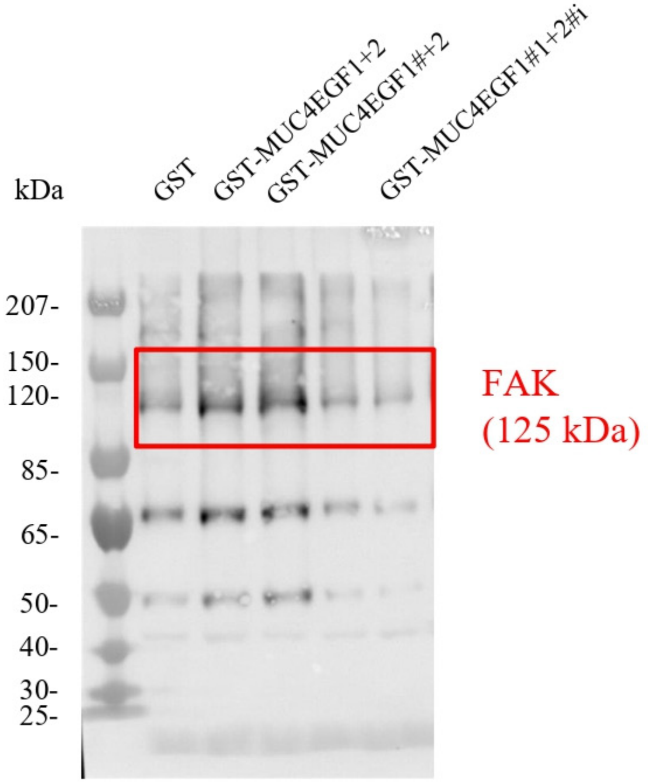

Detail information about Figure 6. FAK (125 kDa) uncropped blot.

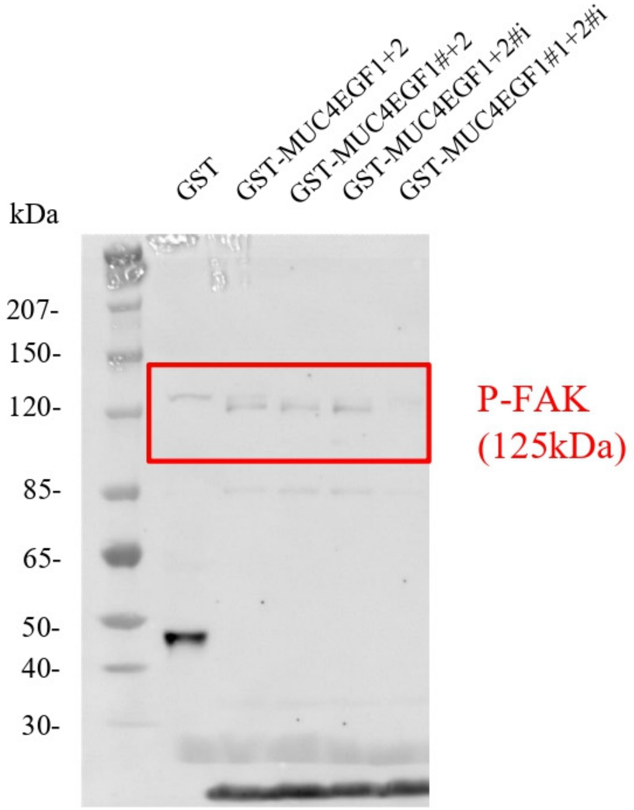

Detail information about Figure 6. Phospho-FAK (125 kDa) uncropped blot.

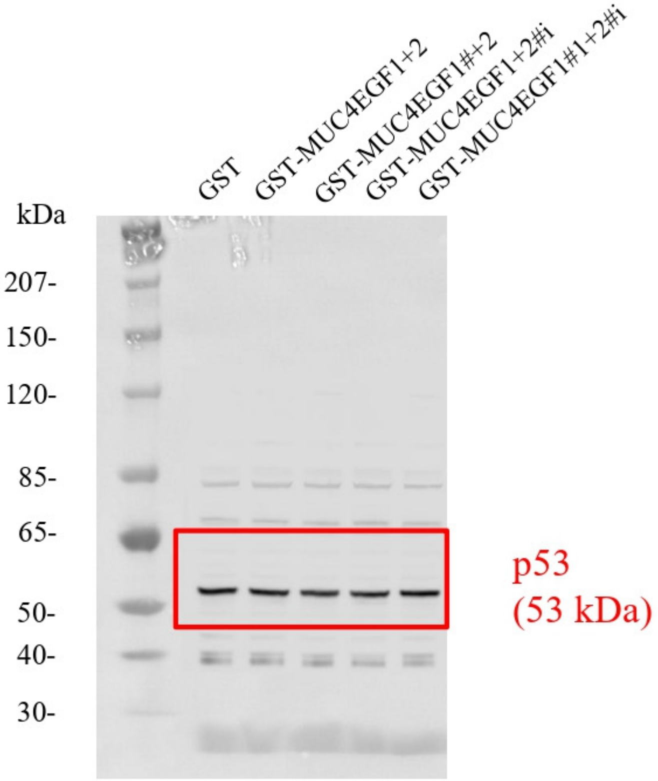

Detail information about Figure 6. p53 (53 kDa) uncropped blot.

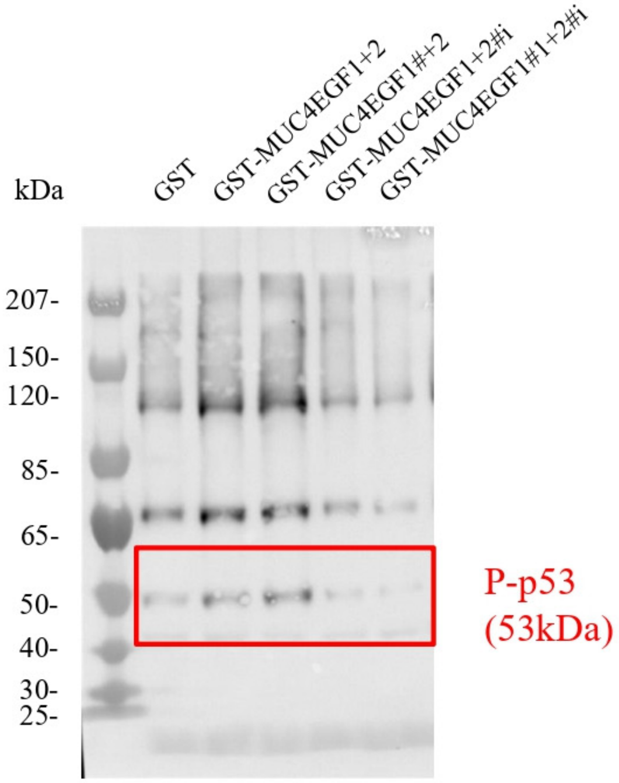

Detail information about Figure 6. Phospho-p53 (53 kDa) uncropped blot.

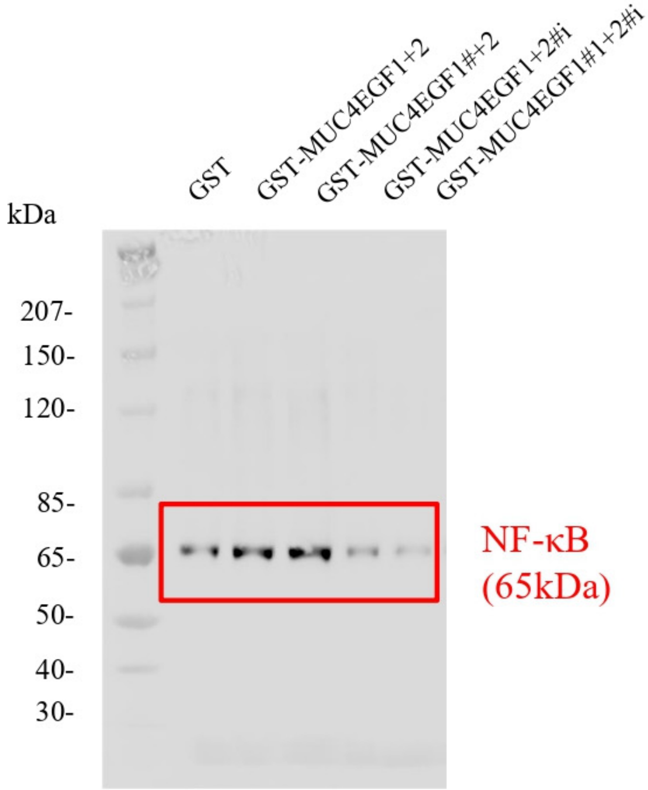

Detail information about Figure 6. NF-κB (65 kDa) uncropped blot.

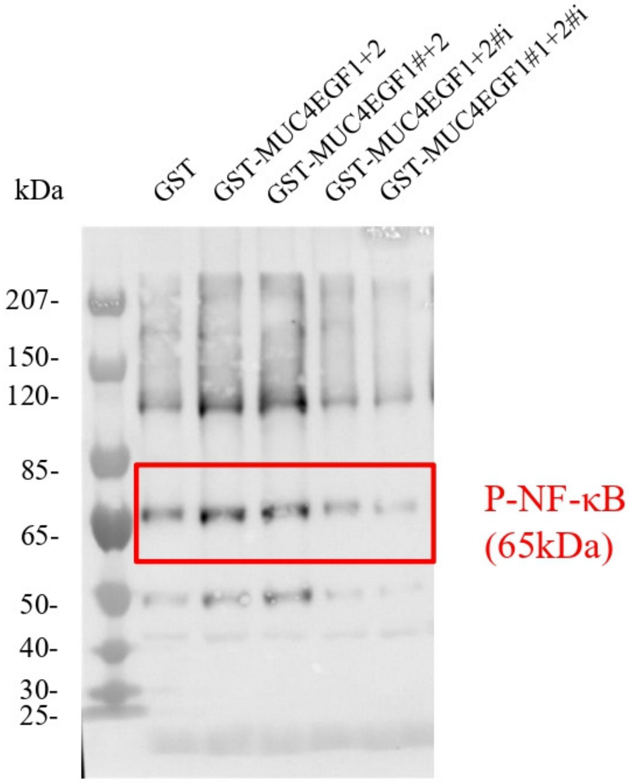

Detail information about Figure 6. Phospho- NF-κB (65 kDa) uncropped blot.

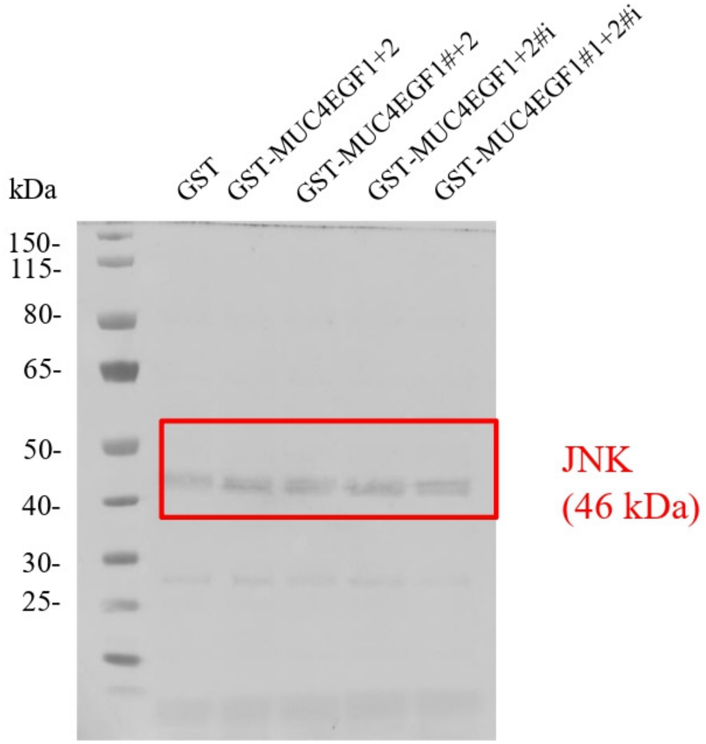

Detail information about Figure 6. JNK (46 kDa) uncropped blot.

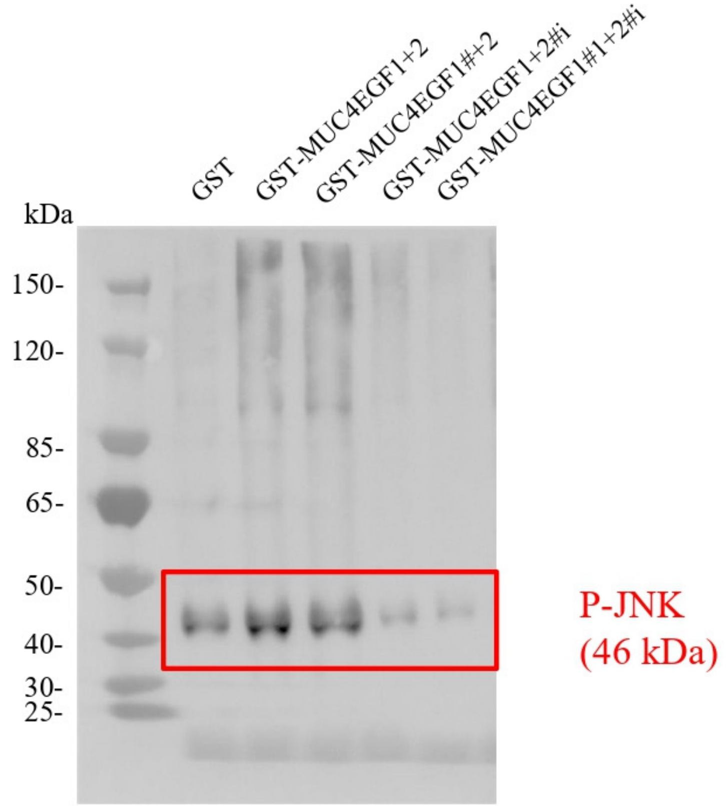

Detail information about Figure 6. Phospho-JNK (46 kDa) uncropped blot.

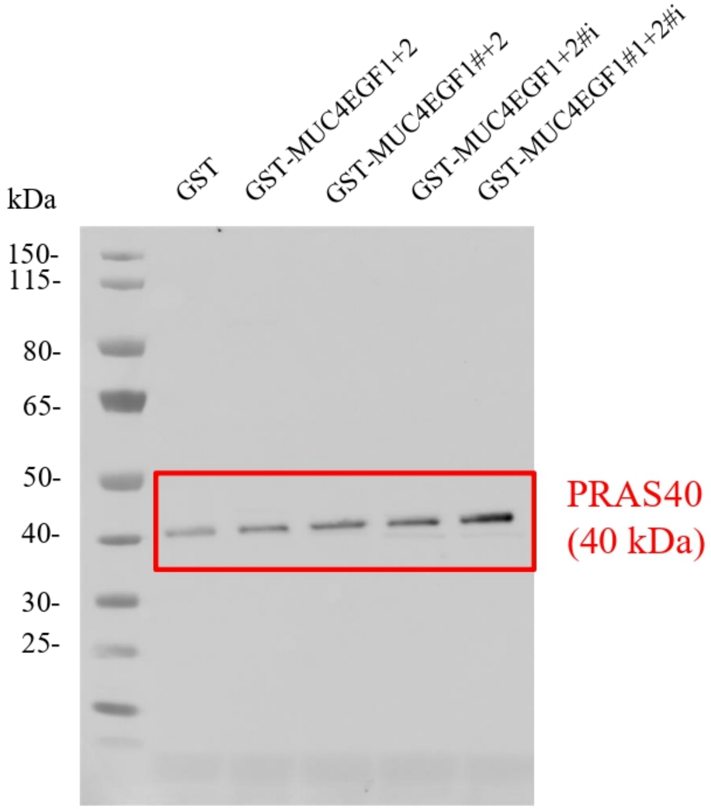

Detail information about Figure 6. PRAS40 (40 kDa) uncropped blot.

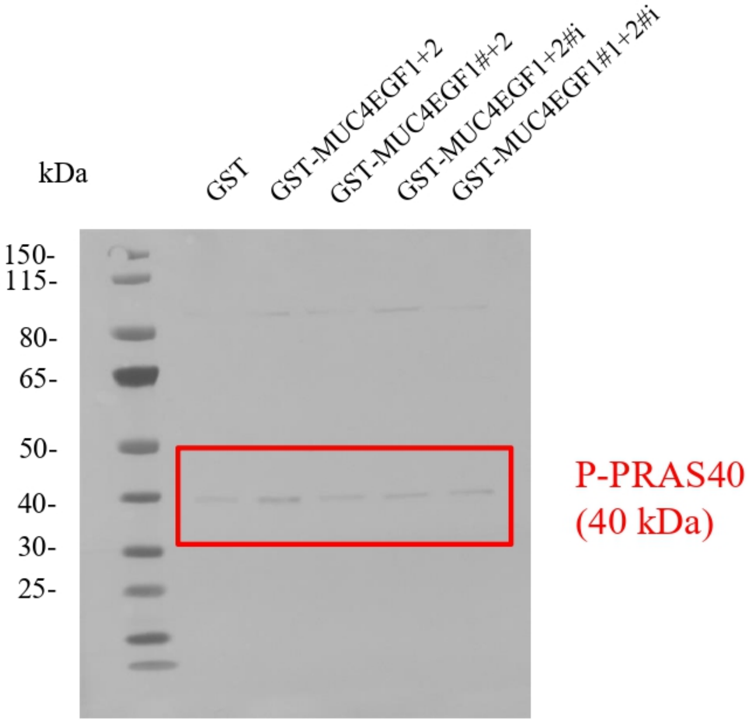

Detail information about Figure 6. Phospho-PRAS40 (40 kDa) uncropped blot.

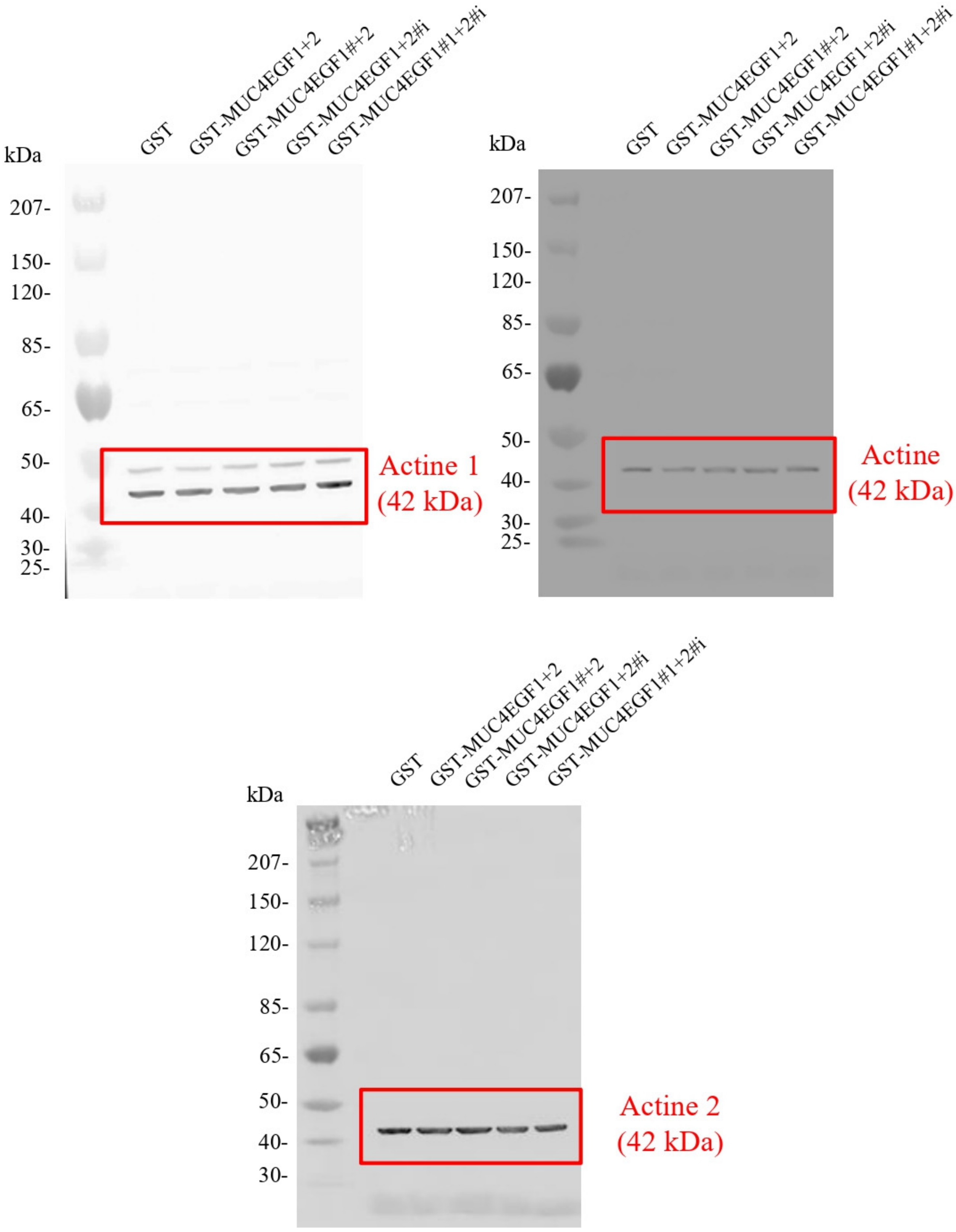

Detail information about Figure 6. Actines (42 kDa) uncropped blots

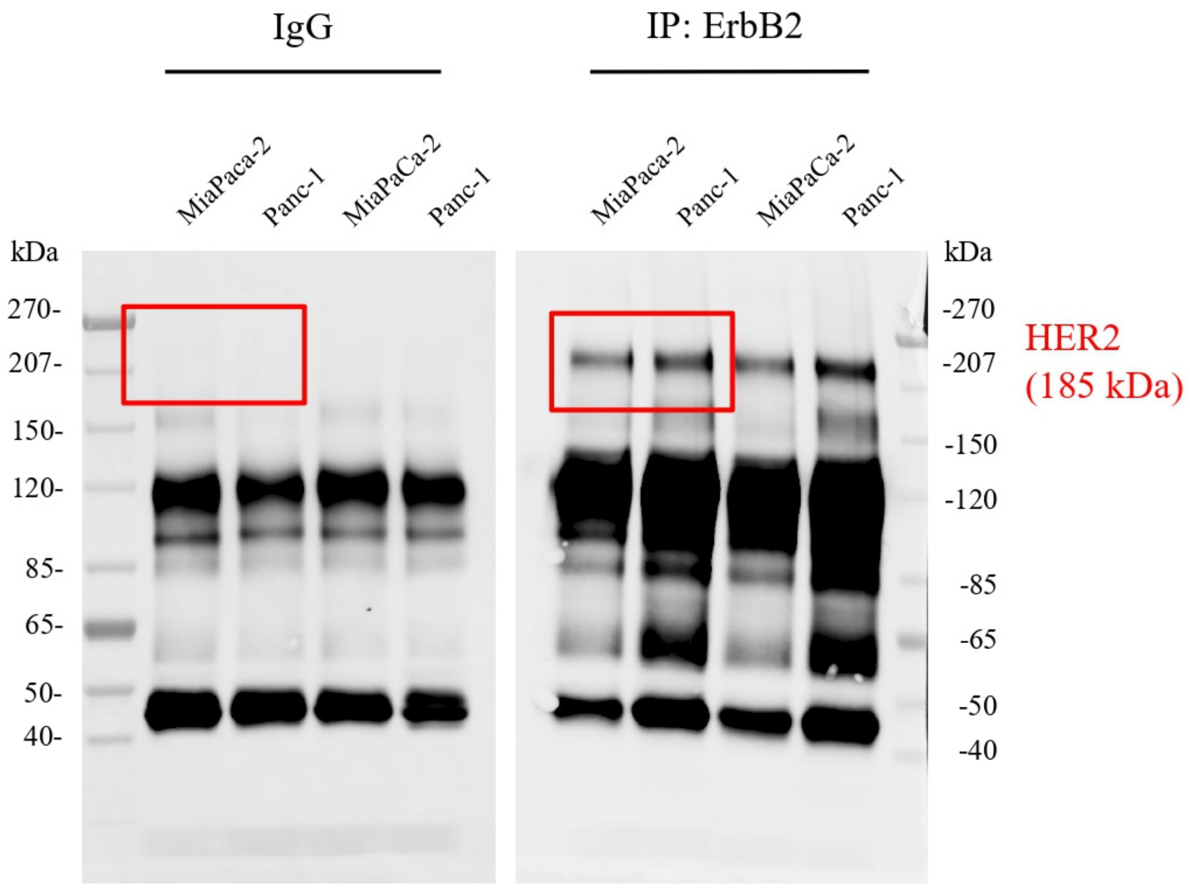

Detail information about Figure S1. Co-immunoprecipitation and immubloting of HER2 (185 kDa) uncropped blots

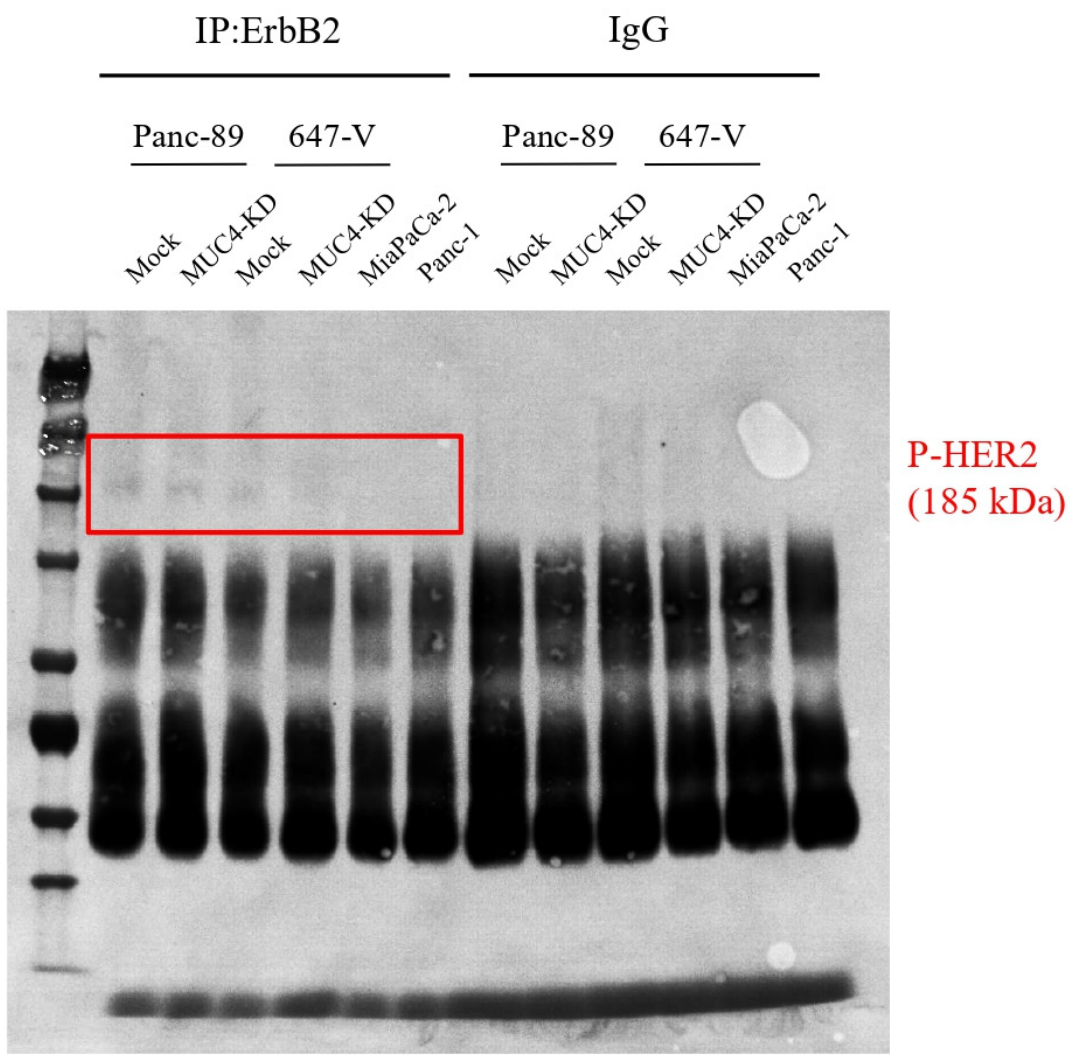

Detail information about Figure S1. Co-immunoprecipitation of HER2 and immubloting of Phospho-HER2 (185 kDa) uncropped blots

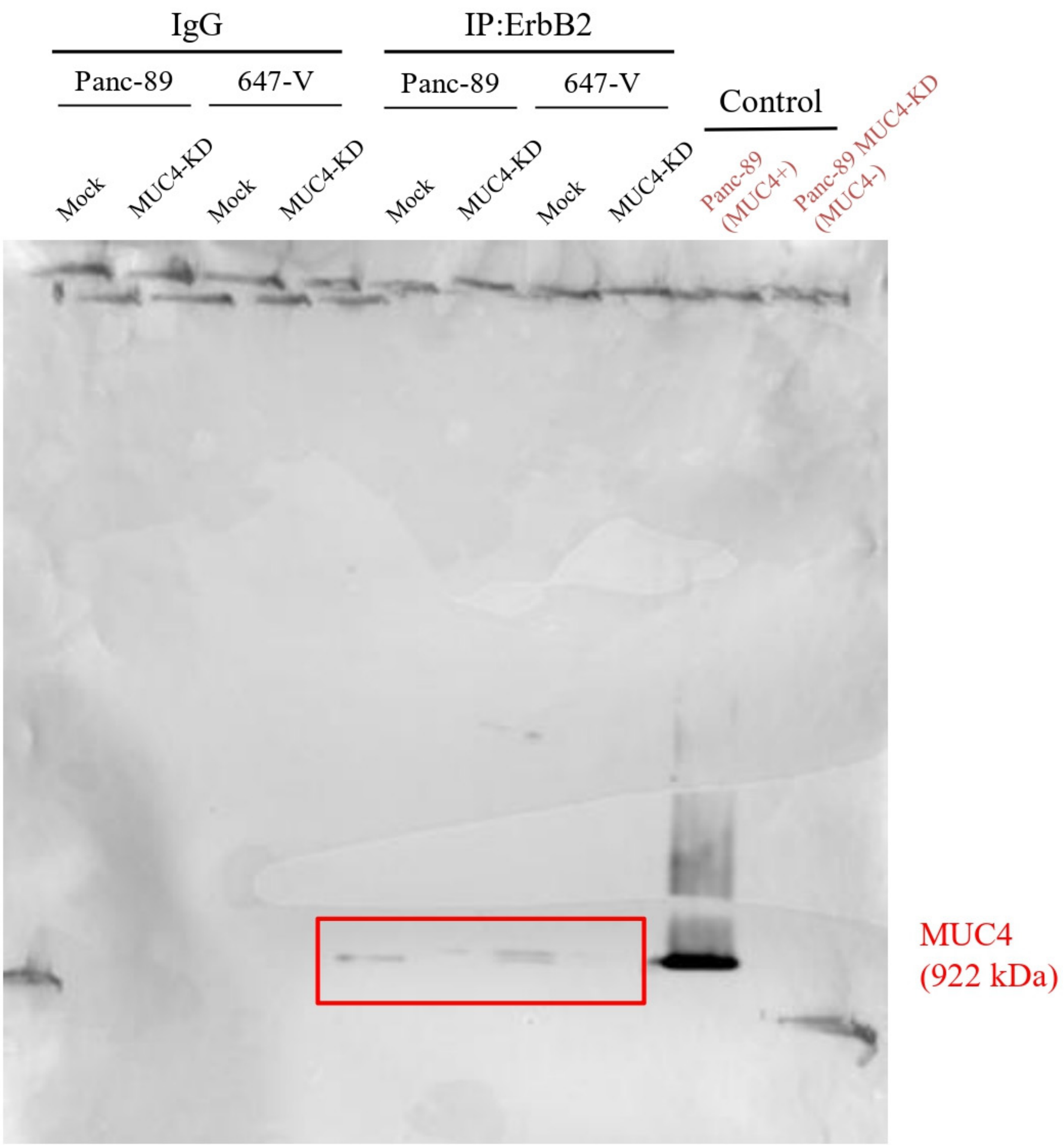

Detail information about Figure S1. Co-immunoprecipitation of HER2 and immubloting of MUC4 (922 kDa) uncropped blots

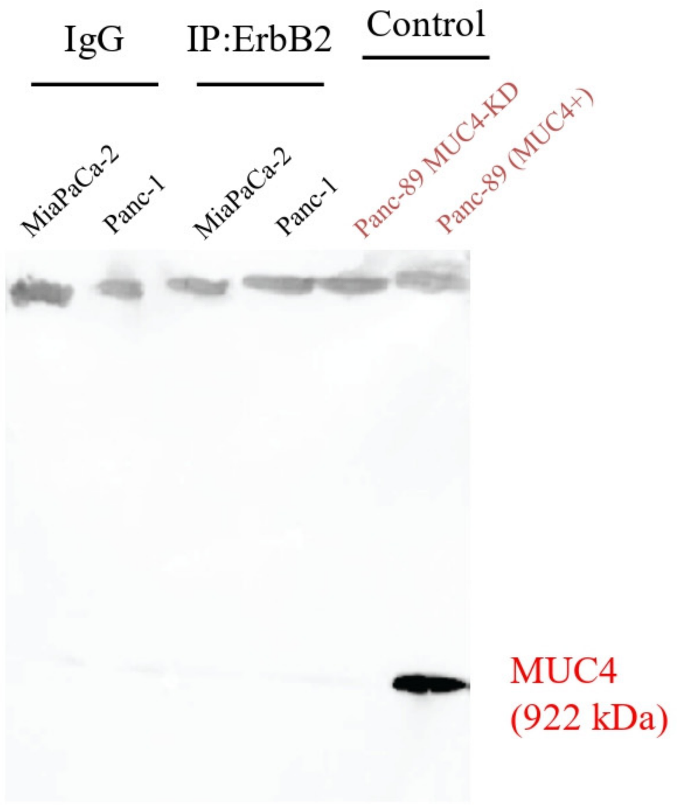

Detail information about Figure S1. Co-immunoprecipitation of HER2 and immubloting of MUC4 (922 kDa) uncropped blots

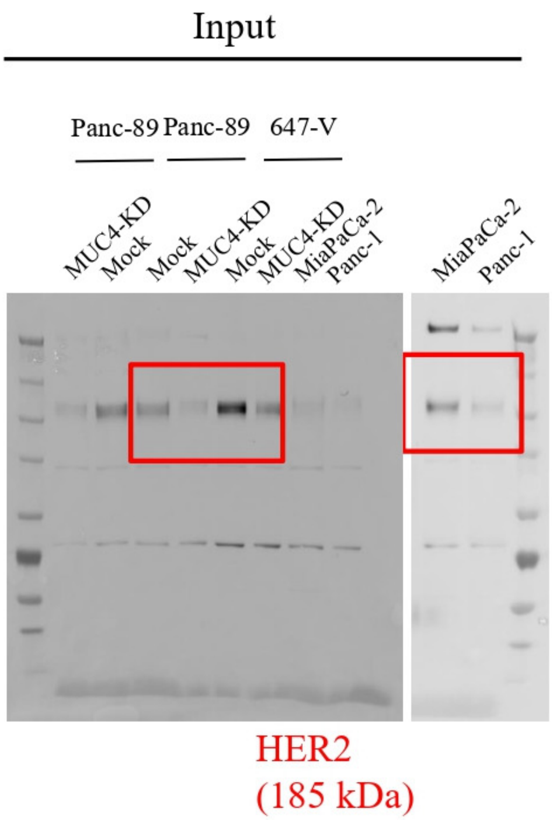

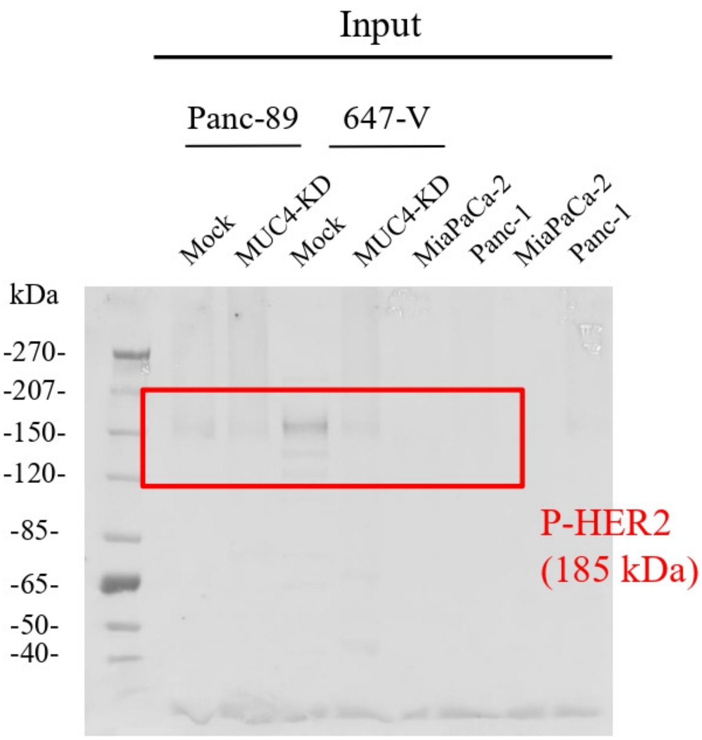

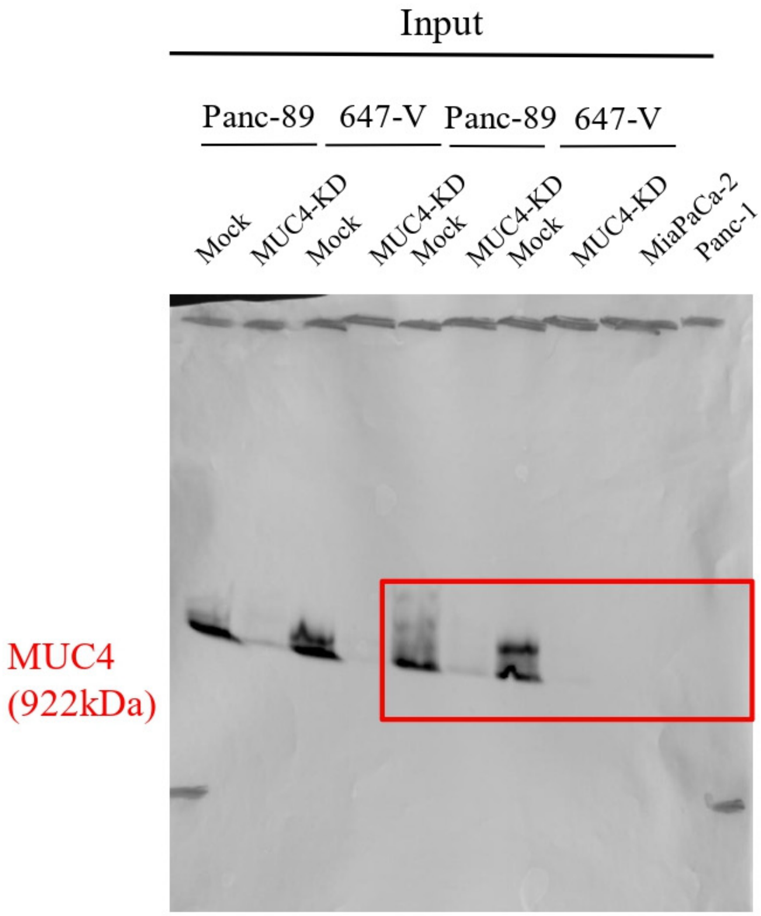

Detail information about Figure S1. Input of HER2 (185kDa), P-HER2 (185kDa) and MUC4 (922 kDa) from the same sample, uncropped blots

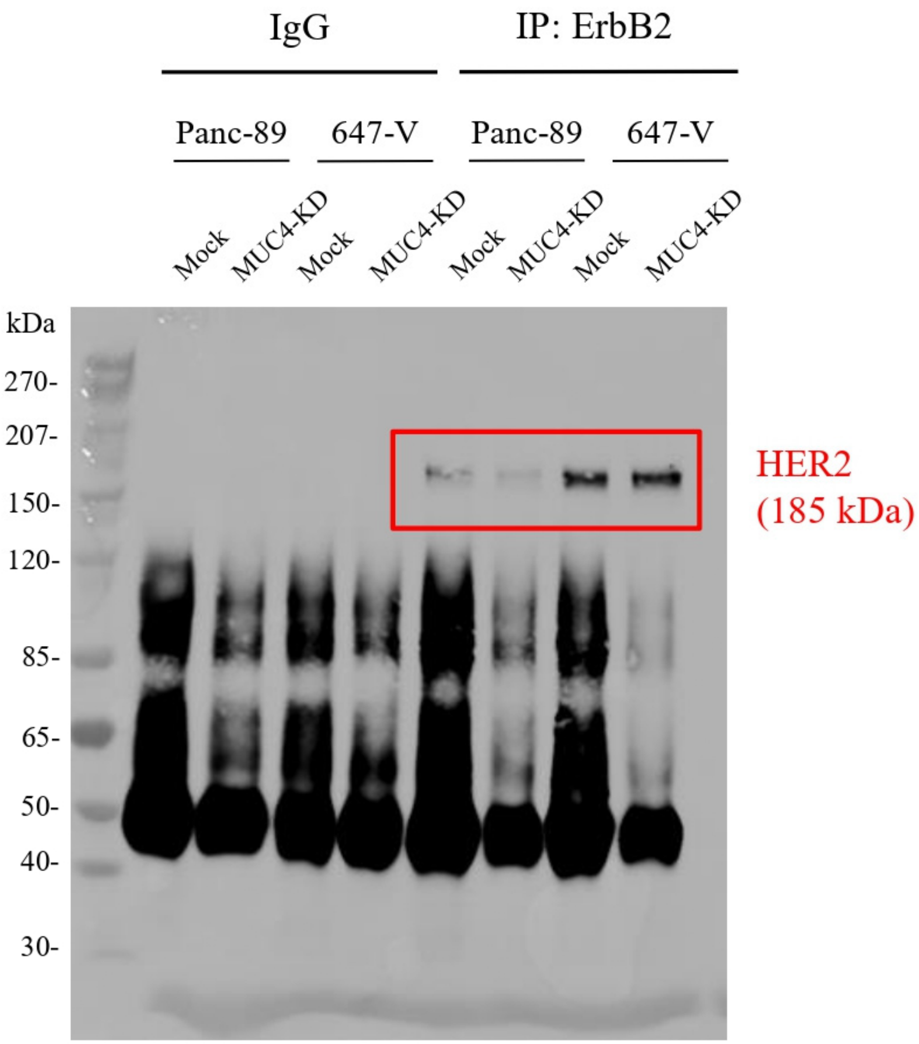

Detail information about Figure S1. Co-immunoprecipitation and immubloting of HER2 (185 kDa) uncropped blots

Figure S13. Original uncropped western blot.
